# Supplementary material for: Comparative efficacy and acceptability of psychotherapies for post-traumatic stress disorder in children and adolescents: study protocol for a systematic review and network meta-analysis
Source: BMJ Open. 2018 Mar 12;8(3):e020198. doi: 10.1136/bmjopen-2017-020198 (PMC5857664; doi:10.1136/bmjopen-2017-020198)
Supplement: Supplementary file 1 [file bmjopen-2017-020198supp001.pdf]

The full search strategy for PubMed

The following explicit search strategy with filters of clinical study will be applied: Condition = [(post-traumatic* or PTSD or PTSS or posttrauma* or trauma* or peritrauma* or peri-trauma* or psychotraumatology or “stress disorder*” or “stress reactions” or “sexually abus*” or “sexual abus*” or “sexually assault*” or “sexual assault*” or “physical abuse*” or “physically abuse*” or “child abuse” or “children abuse” or “natural disaster” or refugee or war or violence or maltreat* or mistreat or acciden* or tsunami* or hurricane* or earthquake or tornado)] AND Intervention = (“trauma focused” or trauma-specific or cogniti* or behavio* or CBT or “eye movement desensitization and reprocessing” or EMDR or EMD or “stress management” or “stress inoculation training” or mindfulness or “art therapy” or “play therapy” or hypnotherapy or biofeedback or counsel* or supportive or interpersonal or bibliotherapy or narrative or narration or exposure* or psychoeducation or “family treatment” or “family therapy” or meditation or relaxation or “problem solving” or “school-based intervention” or “school-based treatment” or “mind-body skills" or “seeking safety” or “mental health” or “writing for recovery” or “psychosocial intervention” or “child centered” or psychodynamic or psychodrama or desensitization or psychological or psychotherap*) AND Age = (adolesc* or boy* or girl* or child* or infant* or juvenil* or minors or pediatri* or paediatri* or pubescen* or puberty or student* or teen* or young or youth* or school* or high-school or preschool* or pre-school* or class*).

The following Medical Subject Headings (MeSH) will also be searched: “Stress Disorders” AND “Psychotherapy” AND (“child” OR“ adolescent”).
