# Supplementary material for: Comparative efficacy and acceptability of psychotherapies for post-traumatic stress disorder in children and adolescents: study protocol for a systematic review and network meta-analysis
Source: BMJ Open. 2018 Mar 12;8(3):e020198. doi: 10.1136/bmjopen-2017-020198 (PMC5857664; doi:10.1136/bmjopen-2017-020198)
Supplement: Author's manuscript [file bmjopen-2017-020198.draft_revisions.pdf]

BMJ Open is committed to open peer review. As part of this commitment we make the peer review history of every article we publish publicly available.

When an article is published we post the peer reviewers' comments and the authors' responses online. We also post the versions of the paper that were used during peer review. These are the versions that the peer review comments apply to.

The versions of the paper that follow are the versions that were submitted during the peer review process. They are not the versions of record or the final published versions. They should not be cited or distributed as the published version of this manuscript.

BMJ Open is an open access journal and the full, final, typeset and author-corrected version of record of the manuscript is available on our site with no access controls, subscription charges or pay-per-view fees (http://bmjopen.bmj.com).

If you have any questions on BMJ Open's open peer review process please email info.bmjopen@bmj.com

BMJ Open

# Comparative Efficacy and Acceptability of Psychotherapies for Post-traumatic Stress Disorder in Children and Adolescents: Study Protocol for a Systematic Review and Network Meta-Analysis


| | |
|---|---|
| Journal: | *BMJ Open* |
| Manuscript ID | bmjopen-2017-020198 |
| Article Type: | Protocol |
| Date Submitted by the Author: | 20-Oct-2017 |


| | |
|---|---|
| Complete List of Authors: | Zhang, Yuqing; The First Affiliated Hospital of Chongqing Medical University, Department of Neurology<br>Zhou, Xinyu; The First Affiliated Hospital of Chongqing Medical University, Department of Psychiatry<br>Yang, Lining; The First Affiliated Hospital of Chongqing Medical University, Department of Neurology<br>Hetrick, Sarah; University of Melbourne, Centre for Youth Mental Health<br>Weisz, John; Harvard University, Department of Psychology<br>Cuijpers, Pim; VU University Amsterdam, Department of Clinical Psychology<br>Barth, Juergen; University Hospital and University of Zurich, Institute for Complementary and Integrative Medicine<br>Del Giovane, Cinzia; University of Modena and Reggio Emilia, Clinical and Diagnostic Medicine and Public Health<br>Yuan, Shuai; The First Affiliated Hospital of Chongqing Medical University, Department of Neurology<br>Cohen, David; Institut des Systèmes Intelligents et de Robotiques, UPMC Univ Paris 06, Department of Child and Adolescent Psychiatry<br>Gillies, Donna; Western Sydney Local Health District, Mental Health<br>Jiang, Xiaofeng; The First Affiliated Hospital of Chongqing Medical University, Department of Neurology<br>Teng, Teng; The First Affiliated Hospital of Chongqing Medical University, Department of Neurology<br>Xie, Peng; The First Affiliated Hospital of Chongqing Medical University, Department of Neurology; Chongqing Medical University, Institute of Neuroscience and the Collaborative Innovation Center for Brain Science |


| | |
|---|---|
| Keywords: | post-traumatic stress disorder, Child & adolescent psychiatry < PSYCHIATRY, psychotherapy, network meta-analysis, systematic review |


SCHOLARONE™
Manuscripts

# Comparative Efficacy and Acceptability of Psychotherapies for Post-traumatic Stress Disorder in Children and Adolescents: Study Protocol for a Systematic Review and Network Meta-Analysis

Yuqing Zhang,[1] Xinyu Zhou,[2] Lining Yang,[1] Sarah E. Hetrick,[3] John R. Weisz,[4] Pim Cuijpers,[5] Jürgen Barth,[6] Cinzia Del Giovane,[7] Shuai Yuan,[1] David Cohen,[8] Donna Gillies,[9] Xiaofeng Jiang,[1] Teng Teng,[1] Peng Xie[1]

[1] Department of Neurology, The First Affiliated Hospital of Chongqing Medical University, Chongqing, China; Institute of Neuroscience and the Collaborative Innovation Center for Brain Science, Chongqing Medical University, Chongqing, China

[2] Department of Psychiatry, The First Affiliated Hospital of Chongqing Medical University, Chongqing, China

[3] Orygen, The National Centre of Excellence in Youth Mental Health, and the Centre of Youth Mental Health, University of Melbourne, Melbourne, Australia

[4] Department of Psychology, Harvard University, Cambridge, MA, USA

[5] Department of Clinical, Neuro and Developmental Psychology, Amsterdam Public Health research institute, Vrije Universiteit Amsterdam, The Netherlands

[6] Institute for Complementary and Integrative Medicine, University Hospital and University of Zurich, Zurich, Switzerland

[7] Department of Diagnostic, Clinical and Public Health Medicine, University of Modena and Reggio Emilia, Modena, Italy

[8] Department of Child and Adolescent Psychiatry, Hôpital Pitié–Salpétrière, Institut des Systèmes Intelligents et Robotiques, Université Pierre et Marie Curie, Paris, France

[9] Western Sydney Local Health District - Mental Health, Westmead, Australia

YZ, XZ and LY contributed equally to this work.

**Direct correspondence to:**

Prof Peng Xie, Department of Neurology, The First Affiliated Hospital of Chongqing Medical University, Yuzhong District, Chongqing, China; E-mail: xiepeng973@126.com.



For peer review only - http://bmjopen.bmj.com/site/about/guidelines.xhtml

Keywords: post-traumatic stress disorder, child, adolescent, psychotherapy, network meta-analysis, systematic review

Word count: 3143.

For peer review only



For peer review only - http://bmjopen.bmj.com/site/about/guidelines.xhtml

**ABSTRACT**

**Introduction**: Post-traumatic stress disorder (PTSD) is common among children and adolescents who are exposed to trauma, and it is often associated with significant negative impacts on their psychosocial functioning and quality of life. Many types of psychotherapies have been found to be effective for PTSD in children and adolescents. However, due to the lack of direct comparisons between different psychotherapies, the hierarchy of treatment efficacy is still unclear. Therefore, we plan to conduct a systematic review and network meta-analysis to evaluate the efficacy and acceptability of various types of psychotherapies for PTSD in children and adolescents.

**Methods and analysis**: A systematic search will be conducted among eight electronic databases, including PubMed, Cochrane, EMBASE, Web of Science, PsycINFO, CINAHL, PILOTS and ProQuest Dissertations, from inception to October 2017. Randomised controlled trials (RCTs), regardless of language, publication year, and publication type, comparing any structured psychotherapies for PTSD to any control condition or alternative treatment in children and adolescents (18 years old or less) will be included. The primary outcome will be efficacy at post-treatment, which is PTSD symptom severity measured by a rating scale reported by the child, parent, or a clinician. The secondary outcomes will include (1) efficacy at follow-up; (2) acceptability (all-cause discontinuation); (3) improvement of anxiety symptoms; (4) improvement of depressive symptoms, and (5) quality of life and functional improvement. Bayesian network meta-analyses for all relative outcome measures will be performed. We will conduct subgroup and sensitivity network meta-analyses to determine whether the findings are affected by study characteristics. The quality of the evidence contributing to network estimates of the primary outcome will be evaluated by the GRADE framework.

**Ethics and dissemination:** No ethical issues are foreseen. The results will be published in a peer–reviewed journal, which will be disseminated electronically and in print. This network meta-analysis may be updated to inform and guide the clinical management of PTSD in children and adolescents.

**Trial registration number:** PROSPERO CRD42016051786.

For peer review only



For peer review only - http://bmjopen.bmj.com/site/about/guidelines.xhtml

**Strengths and limitations of this study**

1. Bayesian network meta-analysis can simultaneously compare various types of treatments by integrating all the best evidence (direct and indirect evidence) to estimate the interrelations across all treatments and establish a treatment hierarchy for psychotherapies for post-traumatic stress disorder in children and adolescents.

2. A number of outcomes will be used to comprehensively assess efficacy at post-treatment and follow-up, acceptability, improvement of anxiety and depressive symptoms, and quality of life and functional improvement.

3. This study will help to guide clinical decision-making regarding the relative efficacy of various types of psychotherapy, and various delivery modalities, in the treatment of post-traumatic stress disorder in children and adolescents.

4. Subgroup and sensitivity network meta-analyses will help to find potential moderators that affect the efficacy of all included psychotherapies.

5. The limitations of included studies will be assessed using the Cochrane Collaboration's Risk of bias' 2.0 tool and the quality of evidence for network estimates of the primary outcome will be evaluated by the GRADE framework.

For peer review only

## BACKGROUND

Trauma is common in children and adolescents. More than two thirds of young people have suffered at least one traumatic event,[1] with approximately 15.9% of whom go on to develop post-traumatic stress disorder (PTSD).[2] As described in the Diagnostic and Statistical Manual of Mental Disorders, 5th edition (DSM-5), PTSD diagnostic criteria includes four symptom clusters: (1) re-experiencing of the traumatic event, (2) avoidance of stimuli associated with the traumatic event, (3) negative alterations in cognition and mood, (4) hyperarousal.[3] Trauma exposure is an essential factor in order to be able to diagnose PTSD, which include physical or sexual abuse, war or terrorism, natural or man-made disasters, witnessing domestic violence, catastrophic illnesses, vehicle or other accidents.[4,5] PTSD is correlated with various adverse consequences for children and adolescents in cognitive, emotional, social, academic, and other functional domains.[6,7] In addition, PTSD commonly co-occurs with other psychiatric comorbidities, such as depressive disorder,[8] anxiety disorder,[8] substance abuse,[9] and ADHD.[10] If children and adolescents do not receive adequate intervention, the symptoms of PTSD may be long-lasting, and have deleterious effects on well-being later in life.[5,11]

Several forms of interventions have been applied to address PTSD symptoms in children and adolescents, from which psychotherapy is the mainstay of treatment.[12] Compared with psychotherapy, medication used in the treatment of PTSD in children and adolescents is limited because (1) the evidence of pharmacological treatments for children and adolescents with PTSD is scant, so that the benefit of medication is not clear;[13] and, (2) medication usage has been associated with a series of safety concerns in children and adolescents, including evidence of a significant increase in the risk of suicidality in children and adolescents being treated with antidepressants.[14,15] Therefore, clinical guidelines recommend that psychotherapy should be used for the initial treatment of PTSD in children and adolescents.[16]

Since the 1990s, psychotherapies have been increasingly applied in the treatment of PTSD in children and adolescents, and the evidence on their efficacy has been reported encouraging in recent reviews.[17-19] Many types of psychotherapies are now available, such as cognitive behavioral therapy (CBT), eye movement desensitization and



For peer review only - http://bmjopen.bmj.com/site/about/guidelines.xhtml

reprocessing (EMDR), behavioral therapy (BT), cognitive therapy, psychodynamic therapy and play therapy. Among these, trauma-focused CBT (TF-CBT), a CBT program that utilises cognitive-behavioral techniques with a trauma-focused component, is the most commonly practiced psychotherapy for children and adolescents with PTSD.[20] TF-CBT has been recommended as a first-line treatment by clinical guidelines for PTSD in children and adolescents.[16,21] However, trauma-focused therapy can be difficult for some therapists to implement,[22,23] and there are some safety concerns in clinical practice (e.g. symptoms worsening and ending treatment prematurely).[24,25] Two recent trials that directly compared TF-CBT with non-TF-CBT reported that both of the two treatment modalities were efficacious.[23,26] Thus, whether the inclusion of a trauma focus component is essential in CBT for PTSD in children and adolescents is still uncertain. Besides, other therapies, such as EMDR and BT, have also been reported effective in treating PTSD symptoms in children and adolescents.[27,28] However, due to the limited number of randomised controlled trials (RCTs) directly comparing different types of psychotherapies, conventional meta-analyses cannot provide a clear answer regarding the best choice for initial treatment, nor with regard to a hierarchy of these psychotherapies.

Network meta-analysis is a newly developed method for evidence synthesis, which is able to integrate direct evidence (from studies directly comparing interventions) with indirect evidence (derived from separate studies addressing a common reference condition) from multiple treatment comparisons to estimate the interrelations across all treatments.[29] This approach enables a simultaneous comparison of multiple treatments without adversely affecting randomisation of treatments within each trial, and can provide hierarchical evidence to guide clinical practice. Using this method, our research group has found a series of important findings among psychotherapies for the treatment of anxiety disorder[30] and depressive disorder[31] in children and adolescents. Therefore, the aim of this paper is to describe the protocol of a Bayesian network meta-analysis that synthesise all the evidence on psychotherapies for PTSD in children and adolescents and provide clinicians with a reliable treatment hierarchy.

**METHODS**



For peer review only - http://bmjopen.bmj.com/site/about/guidelines.xhtml

**Criteria for included studies**

***Types of studies***

All randomised controlled trials (RCTs), including cluster-randomised trials and cross-over trials, will be included. However, only the results from the first randomisation period will be considered in the cross-over trials. For the purpose of reducing heterogeneity between trials, we will exclude quasi-randomised trials (e.g., allocation based on the last number of the date of birth) and trials in which the sample size is less than 10 per study. In addition, to decrease the heterogeneity and inconsistency of this network, trials in which the number of sessions is less than four will be excluded.[32]

***Types of participants***

Studies that enrolled children and adolescents, aged 18 years old or less when they were initially enrolled, will be included in this review. Given that children with significant PTSD symptoms who do not meet full criteria for a PTSD diagnosis often have comparable functional impairment to those with a PTSD diagnosis,[33,34] the clinical guideline suggested that treatment decisions should be based on symptom severity and functional impairment, rather than whether or not they have an actual PTSD diagnosis.[16] Therefore, we will apply the following broad criteria to identify the participants: (1) Full PTSD, as diagnosed according to standardised diagnostic interviews based on international classifications (the Diagnostic and Statistical Manual of Mental Disorders (DSM),[3,35-38] the International Classification of Diseases (ICD) [39,40]) or validated scales for PTSD based on DSM/ ICD criteria;[41-44] (2) Subclinical/Partial PTSD, defined as patients who have experienced psychological trauma and report some subsequent PTSD symptoms in at least one of the four symptom clusters according to DSM-5 (i.e., reexperiencing, avoidance, hyperarousal, negative alterations in cognition and mood);[45] (3) Clinically significant posttraumatic stress symptoms, defined as scoring above a validated cutoff on a PTSD rating scale, such as Kiddie Schedule for Affective Disorders and Schizophrenia (K-SADS)[42], Child PTSD Symptoms Scale (CPSS)[46] and the Impact of Event Scale (IES)[47]. We will include trials in which participants have



For peer review only - http://bmjopen.bmj.com/site/about/guidelines.xhtml

a secondary diagnosis of comorbid general psychiatric disorders, e.g., depressive disorder, anxiety disorder, attention deficit hyperactivity disorder (ADHD) and oppositional defiant disorder (ODD). However, trials in which participants have a diagnosis of acute stress disorder (ASD) or adjustment disorder (AD) will be excluded. Studies where both adults and children/adolescents are included will be eligible for inclusion, if the data for the latter can be obtained separately. Trials conducted in any treatment settings, including outpatient clinics, inpatient services, community clinics and schools will be included.

***Types of interventions***

All RCTs comparing any structured psychotherapy against another structured psychotherapy or any control condition for children and adolescents with PTSD will be included. We will view each of: trauma-focused CBT (TF-CBT), non-trauma-focused CBT (Non-TF-CBT), eye movement desensitization and reprocessing (EMDR), behavioral therapy (BT), cognitive therapy (CT), psychodynamic therapy (DYN), play therapy (PT), and any other structured psychotherapy as independent notes in this network meta-analysis. Trials comparing the same type of psychotherapies, but at different delivery conditions (with or without family involvement), different delivery formats (group, individual, or group plus individual) and different delivery mediums (face-to-face, internet-based) will be considered as the same node in this network meta-analysis. Nevertheless, because TF-CBT is the most commonly studied psychotherapy and recommended by clinical guidelines as first-line choice for children and adolescents with PTSD,[16,21] if data available, we will separate TF-CBT with different delivery conditions, formats, and mediums as independent nodes.

Control conditions can include supportive therapy (ST), waitlist (WL), non-treatment (NT), and treatment as usual (TAU). We will view each of these control conditions as independent nodes in this network meta-analysis. The detailed description of each treatment and control condition are presented in Table 1.

Studies where psychotherapy is used as a combination strategy (e.g., combining psychotherapy with medication) will be excluded, because such designs make it impossible for us to detect effect size of each specific treatment approach.



For peer review only

For peer review only - http://bmjopen.bmj.com/site/about/guidelines.xhtml

***Types of outcome measures***

*Primary outcome*

1. Efficacy at post-treatment, as measured using the end point score from PTSD symptom severity rating scales completed by the child, parent, or a clinician.[48] When end point scores are not reported, we will use change scores (if available).[49] Where more than one scale is reported in a trial, we will extract data from the PTSD symptom scales on a hierarchy, which is based on psychometric properties and appropriateness for use with children and adolescents (Table 2). In addition, where a PTSD symptom scale is reported by different raters in a trial, self-rated outcome will be preferred, then the parent or clinician rated outcome, because self-rated outcome tends to result in conservative effect sizes.[50]

*Secondary outcomes*

1. Efficacy at follow-up, as measured by the score from PTSD scales at the longest point of follow-up (up to 12 months). The selection priority of PTSD scales will be the same as for efficacy at post-treatment. Data from participants who take part in subsequent treatments (e.g., continuing psychotherapy/pharmacotherapy or booster sessions) will be excluded in the follow-up analysis.

2. Acceptability, defined as all-cause discontinuation, as measured by the proportion of patients who discontinued treatment for any cause at the end of treatment. Notably, children and adolescents may discontinue treatment for many different reasons, such as finding it difficult to adhere to long-term treatment, because symptoms worsen, or due to rapid improvement of symptoms.[30]

3. Anxiety symptoms, as measured by the end point score on anxiety symptom severity rating scales. The following scales will be used: Revised Children's Manifest Anxiety Scale (RCMAS), Spence Children's Anxiety Scale (SCAS), Multidimensional Anxiety Scale for Children (MASC), State-Trait Anxiety Inventory for Children (STAIC), Screen for Anxiety and Related Disorders (SCARED). If none of above scales are reported, other valid anxiety scales will be used. When a scale is reported by different raters in a trial, self-rated outcome will be preferred.

4. Depressive symptoms, as measured by the end point score on depressive symptom severity rating scales, The



For peer review only

For peer review only - http://bmjopen.bmj.com/site/about/guidelines.xhtml

following scales will used: Children's Depression Inventory (CDI), Beck depression inventory (BDI), Mood and Feeling Questionnaire (MFQ), Children's Depression Rating Scale Revised (CDRS-R), Hamilton Depression Rating Scale (HAMD). As for anxiety, other valid depression scales will be used if none of the above scales were reported.

5. Quality of life and functional improvement (QoL/functioning). If data are available, we will extract continuous outcomes from scales of quality of life and functional improvement measured at post-treatment. The scales of quality of life include Quality of Life Enjoyment and Satisfaction Questionnaire (Q-LES-Q), Pediatric Quality of Life Inventory (QoL Child Report), the Quality of Life Inventory (QoLI), and others. The scales of functional improvement include the Children's Global Assessment Scale (CGAS), the Global Assessment Functioning (GAF), and others. When both of the scales of quality of life and functional improvement are reported in a trial, we will extract the data of quality of life.

***Search strategy***

We will identify relevant trials from systematic searches in the following electronic databases: PubMed, Cochrane, EMBASE, Web of Science, PsycINFO, CINAHL, PILOTS and ProQuest Dissertations. The timeline will be from database inception to October 2017. The following search strategy with filters of clinical study will be applied: Condition = [(post-traumatic* or PTSD or PTSS or posttrauma* or trauma* or peritrauma* or peri-trauma* or psychotraumatology or "stress disorder*" or "stress reactions" or "sexually abus*" or "sexual abus*" or "sexually assault*" or "sexual assault*" or "physical abuse*" or "physically abuse*" or "child abuse" or "children abuse" or "natural disaster" or refugee or war or violence or maltreat* or mistreat or acciden* or tsunami* or hurricane* or earthquake or tornado)] AND Intervention = ("trauma focused" or trauma-specific or cogniti* or behavio* or CBT or "eye movement desensitization and reprocessing" or EMDR or EMD or "stress management" or "stress inoculation training" or mindfulness or "art therapy" or "play therapy" or hypnotherapy or biofeedback or counsel* or supportive or interpersonal or bibliotherapy or narrative or narration or exposure* or psychoeducation or "family treatment" or "family therapy" or meditation or relaxation or "problem solving" or "school-based intervention" or "school-based treatment" or "mind-body



For peer review only - http://bmjopen.bmj.com/site/about/guidelines.xhtml

skills" or “seeking safety” or “mental health” or “writing for recovery” or “psychosocial intervention” or “child centered” or psychodynamic or psychodrama or desensitization or psychological or psychotherap*) AND Age = (adolesc* or boy* or girl* or child* or infant* or juvenil* or minors or pediatri* or paediatri* or pubescen* or puberty or student* or teen* or young or youth* or school* or high-school or preschool* or pre-school* or class*). The following Medical Subject Headings (MeSH) will also be searched: “Stress Disorders” AND “Psychotherapy” AND (“child” OR“ adolescent”). In addition, international trials registers, such as World Health Organization’s trials portal, ClinicalTrials.gov and Australian New Zealand Clinical Trials Registry (ANZCTR) will be searched for ongoing trials. Furthermore, we will hand-search relevant key psychiatric and medical journals (including *Behavior Therapy, Child Abuse and Neglect, Child Maltreatment, Journal of Counseling and Development, Counseling Outcome Research and Evaluation, Journal of Anxiety Disorders, Journal of Mental Health Counseling, Journal of Trauma Practice, Journal of Traumatic Stress, Journal of the American Academy of Child and Adolescent Psychiatry*). The reference lists of included trials and reviews identified from initial searches will be scanned for more relevant studies. All relevant authors will be contacted to complement the incomplete data.

**Study selection and data extraction**

***Selection of trials***

Two reviewers (YZ and LY) will independently identify potentially eligible studies from the titles and abstracts of records from the search strategies. Studies will be excluded if both reviewers consider that it does not meet eligibility criteria. Then, the full texts of these potentially eligible studies will be reviewed by the same criteria. The inter-rater reliability of the two reviewers will be calculated to detect their consistency. All disagreements will be discussed and resolved by a senior review author (PX or SEH). The references of relevant reviews and included trials will be checked by YZ and LY. Where multiple publications derive from a common data set, we will select the trials in which the relevant outcomes we predefined in this protocol were reported completely. We will report the reasons for exclusion for each trial



For peer review only - http://bmjopen.bmj.com/site/about/guidelines.xhtml

in the characteristics of excluded studies list. Finally, a flow chart will be used to present the process of trial screening in this meta-analysis.

***Risk of bias assessment***

Two reviewers (YZ and XZ) will independently assess the methodological quality of the included studies. According to the Cochrane Collaboration's Risk of bias' 2.0 tool,[51] the risk of bias will be rated as "low risk", "high risk", or "some concerns" in the following domains: (1) bias arising from the randomization process, (2) bias due to deviations from intended interventions, (3) bias due to missing outcome data, (4) bias in measurement of the outcome, (5) bias in selection of the reported result, (6) overall bias. The inter-rater reliability of the two reviewers assessing the risk of bias will also be calculated. Any disagreements will be resolved by a senior review author (PX or SEH).

***Data extraction***

A standardised data extraction form will be used by two independent reviewers (YZ and XZ) to record the relevant parameters from the original paper, including study characteristics (e.g., title, first author, publication year, publication type, publication journal, location, and sponsor), patient characteristics (e.g., diagnostic criteria for PTSD, type of trauma, severity of PTSD symptoms, comorbidities, and the number, mean age and gender of participants), intervention details (e.g., type of psychotherapy, treatment format, treatment setting, treatment duration, the number of sessions, follow-up duration, and co-interventions) and outcome measures (mean scores, number of participants, and outcome raters for each pre-defined outcome). A table will be used to present the main characteristics of each study included in this review. The reliability of data extraction from the reviewers will also be assessed. Any disagreements will be resolved by a senior review author (PX or SEH).

**Statistical analysis**

We will perform Bayesian network meta-analysis for each outcome with random-effects model in WinBUGS (version 1.4.3). In addition, as a reference for relative outcomes of network meta-analyses, conventional pairwise



For peer review only

For peer review only - http://bmjopen.bmj.com/site/about/guidelines.xhtml

meta-analyses will also be performed for each outcome with a random-effects model in Stata (version 13.0). Standardised mean difference (SMD, Cohen's d) will be used as a measure of effect size where outcome is measured on different scales. For dichotomous outcomes, the effect sizes will be calculated as odds ratios (OR). Statistical heterogeneity in each pairwise comparison will be assessed with the $I^2$ statistic and p value.[52] When the mean values or SDs of relative outcomes are missing, we will obtain such value by conversion from p-values, t-values, confidence intervals or standard errors.[53] Missing continuous outcome data will be analysed using the completer data.

The pooled estimates of network meta-analysis will be obtained using the Markov Chains Monte Carlo method. Two Markov chains will be run simultaneously with different arbitrarily chosen initial values. Trace plots and the Brooks-Gelman-Rubin statistic will be monitored to ensure convergence.[54] When the model is adequately convergent, the foregoing samples will be discarded. Then 100,000 subsequent simulations will be run as the posterior summaries. All results will be reported as effect sizes (SMD or OR) and their 95% credible intervals (CrI). We will assume a common heterogeneity parameter for each comparison and assess the global heterogeneity using the $I^2$ statistic with 'gemtc' package in R (version 3.2.2). Furthermore, the inconsistency between direct and indirect evidence will be evaluated, which will include the assessment of global inconsistency (by comparing the fit and parsimony of consistency and inconsistency models), local inconsistency (by calculating the difference among direct and indirect evidence in closed loops in the network)[55] and the inconsistency calculated by node splitting method (by assessing the difference between direct and indirect estimates within a particular comparison).[56] Probability values will be summarised and reported as surface under the cumulative ranking curve (SUCRA) to provide a hierarchy of the treatments.[57]

***Subgroup analysis***

Where possible, we will conduct network meta-regression of data on primary outcome, to evaluate the influence of the following potential moderators: (1) age group; (2) sex ratio; (3) number of sessions; (4) sample size; (5) risk of bias; (6) trauma types (acute/single trauma vs chronic trauma); (7) diagnosis criteria (youth with a diagnosis vs with



For peer review only - http://bmjopen.bmj.com/site/about/guidelines.xhtml

subsyndromal symptoms); (8) source of outcome measure (self-rated vs observer-rated). If the data are insufficient to conduct subgroup analyses for some moderators, we will perform sensitivity analyses by omitting specific trials from the overall analysis.

***Other analyses***

Comparison-adjusted funnel plots and Egger's test will be used for each outcome to examine whether there is dominant publication bias exist in this network meta-analysis.[56] In addition, we will evaluate the quality of evidence for primary outcome by using the GRADE framework, which characterises the quality of a body of evidence on the basis of the study limitations, imprecision, inconsistency, indirectness, and publication bias for network estimates.[58]

**Ethics and dissemination**

This network meta-analysis will use data in the public domain. Therefore, no formal ethical assessment and informed consent are required. The results will be published in a peer-reviewed journal and disseminated electronically and in print.



For peer review only - http://bmjopen.bmj.com/site/about/guidelines.xhtml

**Contributors**

PX, YZ and XZ conceived the study and drafted the manuscript. SEH, JRW, PC, JB, CDG, DC, DG assisted in protocol design and revision. YZ, XZ, LY, SY, XJ, and TT participated in the search strategy development, and will carry out the most work of study selection, risk of bias assessment, and data collection. PX, and SEH will help to revolve the disagreements and check the data. CDG, YZ and XZ participated in the design of data synthesis and analysis, and will conduct the statistical analyses. All the authors have approved the publication of the protocol.

**Funding** This work is supported by National Key Research and Development Programm of China (Grant No.2017YFA0505700). The funders had no role in the protocol design; the writing of the protocol; or the decision to submit the protocol for publication.

**Competing interests** SEH is an editor of the Cochrane Common Mental Disorders Group, and an author on the Cochrane systematic review on treatments for PTSD in young people. During the last two years, David Cohen reported past consultation for or the receipt of honoraria from Otsuka, Shire, Lundbeck and Roche. DG is the primary author of two reviews of psychological therapies for children and adolescents who were diagnosed with PTSD or exposed to trauma. YZ, XZ, LY, JRW, PC, JB, CDG, SY, XJ, TT, and PX declare no competing interests.

**Provenance and peer review** Not commissioned; externally peer-reviewed.

**Open Access** This is an Open Access article distributed in accordance with the Creative Commons Attribution- Non Commercial (CC BY-NC 4.0) license, which permits others to distribute, remix, adapt, build upon this work non-commercially, and license their derivative works on different terms, provided the original work is properly cited and the use is non-commercial. See: http:// creativecommons.org/- licenses/by-nc/4.0/



For peer review only - http://bmjopen.bmj.com/site/about/guidelines.xhtml

1 Copeland WE, Keeler G, Angold A, et al. Traumatic events and post-traumatic stress in childhood. *Arch General Psychiatry* 2007;64:577–84.

2. Alisic E, Zalta AK, Van Wesel F, et al. Rates of post-traumatic stress disorder in trauma-exposed children and adolescents: meta-analysis. *Br J Psychiatry* 2014;204:335-40

3. American Psychiatric Association. Diagnostic and statistical manual of mental disorders. 5th edn. Arlington, VA: American Psychiatric Publishing, 2013.

4. Donnelly CL. Amaya-Jackson L. Post-traumatic stress disorder in children and adolescents: epidemiology, diagnosis and treatment options. *Paediatr Drugs* 2002;4:159-70.

5. Taylor TL, Chemtob CM. Efficacy of treatment for child and adolescent traumatic stress. *Arch Pediatr Adolesc Med* 2004;158:786-91.

6. Dorsey S, McLaughlin KA, Kerns SEU, et al. Evidence Base Update for Psychosocial Treatments for Children and Adolescents Exposed to Traumatic Events. *J Clin Child Adolesc Psychol* 2017;46:303-30.

7. De Bellis MD, Hooper SR, Woolley DP, et al. Demographic, maltreatment, and neurobiological correlates of PTSD symptoms in children and adolescents. *J Pediatr Psychol* 2010;35:570-7.

8. Dixon A, Howie P, Starling J. Trauma exposure, posttraumatic stress, and psychiatric comorbidity in female juvenile offenders. *J Am Acad Child Adolesc Psychiatry* 2005;44:798-806.

9. Lipschiz D, Rasmusson AM, Anyan W, et al. Posttraumatic stress disorder and substance use in inner city girls. *J Nervous Mental Disease* 2003;191:714-21.

10. Ford JD, Racusin R, Ellis CG, et al. Child maltreatment, other trauma exposure and posttraumatic symptomatology among children with oppositional disorder and attention deficit hyperactivity disorders. *Child Maltreat* 2000;5:205-17.

11. Gerson R, Rappaport N. Traumatic stress and posttraumatic stress disorder in youth: recent research findings on



For peer review only - http://bmjopen.bmj.com/site/about/guidelines.xhtml

clinical impact, assessment, and treatment. *J Adolesc Health* 2013;52:137–43.

12. Keeshin BR, Strawn JR. Psychological and pharmacologic treatment of youth with posttraumatic stress disorder: an evidence-based review. *Child Adolesc Psychiatr Clin N Am* 2014;23:399-411,

13. Bajor LA, Ticlea AN, Osser DN. The Psychopharmacology Algorithm Project at the Harvard South Shore Program: an update on posttraumatic stress disorder. *Harv Rev Psychiatry* 2011;19:240-58.

14. U.S. Food and Drug Administration (FDA). Suicidality in children and adolescents being treated with antidepressant medications. http://www.fda.gov/drugs/drugsafety/postmarketdrugsafetyinformationforpatientsandproviders/ ucm161679.htm (accessed 2 Aug 2017).

15. Cipriani A, Zhou X, Del Giovane C, et al. Comparative efficacy and tolerability of antidepressants for major depressive disorder in children and adolescents: a network meta-analysis. *Lancet* 2016;388:881-90.

16. Cohen JA, Bukstein O, Walter H, et al. Practice Parameter for the assessment and treatment of children and adolescents with posttraumatic stress disorder. *J Am Acad Child Adolesc Psychiatry* 2010;49:414–30.

17. Gillies D, Taylor F, Gray C, et al. Psychological therapies for the treatment of post-traumatic stress disorder in children and adolescents. *Cochrane Database Syst Rev* 2012;12:CD006726.

18. Gillies D, Maiocchi L, Bhandari AP, et al. Psychological therapies for children and adolescents exposed to trauma. *Cochrane Database Syst Rev* 2016;10:CD012371.

19. Gutermann J, Schreiber F, Matulis S, et al. Psychological treatments for symptoms of posttraumatic stress disorder in children, adolescents, and young adults: a meta-analysis. *Clin Child Fam Psychol Rev* 2016;19:77-93.

20. Lenz AS, Hollenbaugh KM. Meta-analysis of trauma-focused cognitive behavioral therapy for treating PTSD and co-occurring depression among children and adolescents. *Counseling Outcome Research and Evaluation* 2015;6:18-32.

21. National Collaborating Centre for Mental Health (NCCMH). Post-traumatic stress disorder: The management of



For peer review only - http://bmjopen.bmj.com/site/about/guidelines.xhtml

PTSD in adults and children in primary and secondary care. National Clinical Practice Guideline Number 26. London: National Institute for Clinical Excellence (NICE), 2005.

22. Cohen J, Mannarino AP, Deblinger E. Treating Trauma and Traumatic Grief in Children and Adolescents. New York: Guilford; 2006.

23. Salloum A, Overstreet S. Grief and trauma intervention for children after disaster: exploring coping skills versus trauma narration. *Behav Res Ther* 2012;50:169-79.

24. Linden M, Schermuly-Haupt ML. Definition, assessment and rate of psychotherapy side effects. *World Psychiatry* 2014;13:306-9.

25. Nutt DJ, Sharpe M. Uncritical positive regard? Issues in the efficacy and safety of psychotherapy. *J Psychopharmacol* 2008;22:3-6.

26. Deblinger E, Mannarino AP, Cohen JA, et al. Trauma-focused cognitive behavioral therapy for children: impact of the trauma narrative and treatment length. *Depress Anxiety* 2011;28:67-75.

27. Ahmad A, Larsson B, Sundelin-Wahlsten V. EMDR treatment for children with PTSD: results of a randomized controlled trial. *Nord J Psychiatry* 2007;61:349-54.

28. Ruf M, Schauer M, Neuner F, et al. Narrative exposure therapy for 7- to 16-year-olds: A randomized controlled trial with traumatized refugee children. *J Trauma Stress* 2010;23:437-45.

29. Mavridis D, Giannatsi M, Cipriani A, et al. A primer on network meta-analysis with emphasis on mental health. *Evid Based Ment Health* 2015;18:40-6.

30. Zhang Y, Zhou X, James AC, et al. Comparative efficacy and acceptability of psychotherapies for acute anxiety disorders in children and adolescents: study protocol for a network meta-analysis. *BMJ Open* 2015;5:e008572.

31. Zhou X, Hetrick SE, Cuijpers P, et al. Comparative efficacy and acceptability of psychotherapies for depression in children and adolescents: a systematic review and network meta-analysis. *World Psychiatry* 2015;14:207-22.



For peer review only

For peer review only - http://bmjopen.bmj.com/site/about/guidelines.xhtml

32. National Collaborating Centre for Mental Health (UK). Post-traumatic stress disorder: The management of PTSD in adults and children in primary and secondary care. Gaskell, 2005.

33. Carrion VG, Weems CF, Ray R, et al. Toward an empirical definition of pediatric PTSD: the phenomenology of PTSD in youth. *J Am Acad Child Adolesc Psychiatry* 2002;41:166-73.

34. Scheeringa M, Zeanah C, Myers L, et al. Predictive validity in a prospective follow-up of PTSD in preschool children. *J Am Acad Child Adolesc Psychiatry* 2005;44:899-906.

35. American Psychiatric Association (APA). Diagnostic and statistical manual of mental disorders (DSM-III). 3rd edn. Washington DC: American Psychiatric Association, 1980.

36. American Psychiatric Association (APA). Diagnostic and statistical manual of mental disorders (DSM-III-R). 3rd revised edn. Washington DC: American Psychiatric Association, 1987.

37. American Psychiatric Association (APA). Diagnostic and statistical manual of mental disorders (DSM-IV). 4th edn. Washington DC: American Psychiatric Association, 1994.

38. Americ an Psychiatric Association (APA). Diagnostic and statistical manual of mental disorders (DSM-IV-TR). 4th edn. Washington DC: American Psychiatric Association, 2000.

39. World Health Organization (WHO). The ninth revision of the international classification of diseases and related health problems (ICD-9). Geneva: World Health Organization, 1978.

40. World Health Organization (WHO). The tenth revision of the international classification of diseases and related health problems (ICD-10). Geneva: World Health Organization, 1992.

41. Nader KO, Newman E, Weathers FW, et al. Clinician-Administered PTSD scale for children and adolescents for DSM-IV (CAPS-CA). Los Angeles, USA: National Center for PTSD & UCLA Trauma Psychiatry Program collaboration, 1998.

42. Pynoos RS, Rodriguez N, Steinberg A, et al. The UCLA PTSD Reaction Index for DSM-IV (Revision 1). Los

For peer review only



For peer review only - http://bmjopen.bmj.com/site/about/guidelines.xhtml

Angeles: UCLA Trauma Psychiatry Program, 1998.

43. Kaufman J, Birmaher B, Brent DA, et al. Schedule for affective disorders and schizophrenia for school-aged children: present and lifetime version (K-SADS-PL): initial reliability and validity data. *J Am Acad Child Adolesc Psychiatry* 1996;36:980–88.

44. Silverman WK, Albano AM. Anxiety Disorders Interview Schedule for DSM-IV: child and parent versions. San Antonio, TX: Psychological Corporation, 1996.

45. Franklin CL, Piazza V, Chelminski I, et al. Defining subthreshold PTSD in the DSM-IV literature: a look toward DSM-5. *J Nerv Ment Dis* 2015;203:574-7.

46. Foa EB, Johnson KM, Feeny NC, et al. The child PTSD symptom scale: a preliminary examination of its psychometric properties. *J Clin Child Psychol* 2001;30:376-84.

47. Horowitz M, Wilner N, Alvarez W. Impact of event scale: a measure of subjective stress. *Psychosom Med* 1979;41:209–18.

48. Cuijpers P, Weitz E, Cristea IA, Twisk J. Pre-post effect sizes should be avoided in meta-analyses. *Epidemiol Psychiatr Sci* 2017;26:364-8.

49. da Costa BR, Nüesch E, Rutjes AW, et al. Combining follow-up and change data is valid in meta-analyses of continuous outcomes: a meta-epidemiological study. *J Clin Epidemiol* 2013;66:847–55.

50. Cuijpers P, Li J, Hofmann SG, et al. Self-reported versus clinician-rated symptoms of depression as outcome measures in psychotherapy research on depression: a meta-analysis. *Clin Psychol Rev* 2010;30:768–78.

51. Higgins JPT, Sterne JAC, Savović J, et al. A revised tool for assessing risk of bias in randomized trials In: Chandler J, McKenzie J, Boutron I, Welch V (editors). Cochrane Methods. Cochrane Database of Systematic Reviews 2016, Issue 10 (Suppl 1). dx.doi.org/10.1002/14651858. CD201601

52. Higgins JPT, Thompson SG, Deeks JJ, et al. Measuring inconsistency in meta-analyses. *BMJ* 2003;327:557–60.



For peer review only

For peer review only - http://bmjopen.bmj.com/site/about/guidelines.xhtml

53. Furukawa TA, Barbui C, Cipriani A, et al. Imputing missing standard deviations in meta-analyses can provide accurate results. *J Clin Epidemiol* 2006;59:7–10.

54. Brooks SP, Gelman A. Alternative methods for monitoring convergence of iterative simulations. J *Comput Graph Stat* 1998;7:434–45.

55. Chaimani A, Higgins JP, Mavridis D, et al. Graphical tools for network meta-analysis in STATA. *PLoS One* 2013;8:e76654.

56. Dias S, Welton NJ, Caldwell DM, et al. Checking consistency in mixed treatment comparison meta-analysis. *Stat Med* 2010;29:932–44.

57. Salanti G, Ades AE, Ioannidis JP. Graphical methods and numerical summaries for presenting results from multiple-treatment meta-analysis: an overview and tutorial. *J Clin Epidemiol* 2011;64:163–71.

58. Salanti G, Del Giovane C, Chaimani A, et al. Evaluating the quality of evidence from a network meta-analysis. *PLoS One* 2014;9:e99682.

For peer review only



For peer review only - http://bmjopen.bmj.com/site/about/guidelines.xhtml

**Tables**

**Table 1 Description of Psychotherapeutic interventions and control condition**

| Interventions | Abbreviation | Description |
|---|---|---|
| ***Psychotherapeutic Intervention:*** | | |
| Trauma-focused cognitive-behavioral therapy | TF-CBT | CBT is a combination of cognitive and behavioral techniques. It also involves additional techniques such as relaxation training, affective modulation skills, and enhancement of future safety and development. TF-CBT is a CBT program that involves a trauma focus, which is usually performed through exposure or cognitive processing of thoughts related to the trauma. |
| Non-trauma-focused cognitive-behavioral therapy | Non-TF-CBT | Non-TF-CBT is a CBT program that focuses on teaching skills for the reduction of anxiety. These treatments utilise procedures that directly target the person's beliefs and behaviors rather than the discussions of specific traumas. |
| Cognitive therapy | CT | CT mainly uses cognitive restructuring training, which aims at examining youths' automatic thoughts and core schemas and evaluating the accuracy and affective consequences of their views. They aim to teach youths to engage in "rational" thinking about themselves, the traumatic incident, and the world. |
| Behavioral therapy | BT | BT uses some form of behavioral training, especially for exposure -based therapy and narrative therapy, to help youth reduce trauma-related symptoms. BT is based on principles of habituation. |
| Eye movement desensitization and reprocessing | EMDR | EMDR aims to help a person reprocess their memories of a traumatic event. The therapy involves bringing distressing trauma-related images, beliefs, and bodily sensations to mind. |
| Psychodynamic therapy | DYN | Psychodynamic psychotherapy focuses on integrating the traumatic experience into the life experience of the individual as a whole. Childhood issues are often felt to be important. |
| Play therapy | PT | PT used techniques to engage participants in recreational activities to help them cope with their problems and fears. |
| Stress management | SM | SM mainly includes some form of relaxation or biofeedback |
| ***Control conditions:*** | | |
| Supportive therapy | ST | ST is an unstructured therapy without specific psychological techniques that it helped people to ventilate their experiences and emotions and offering empathy. e.g. supportive counseling, attention control, minimal contact, active listening, common factor control, nonspecific control |
| Treatment-as-usual | TAU | TAU is often described as "usual care" or "usual community treatment" in trials, which is not considered to be an unstructured intervention but may have some treatment effects. |
| Waitlist | WL | WL is a control condition in which the participants receive no active treatment during the study but are informed that they can receive one after the study period is over. |
| No-treatment | NT | NT is a control condition in which the participants receive no active treatment during the study and in which they do not expect to receive such after the study is over. |



For peer review only

For peer review only - http://bmjopen.bmj.com/site/about/guidelines.xhtml

**Table 2 Hierarchy of PTSD symptom severity measurement scales**

| Hierarchy | PTSD symptom severity rating scales | Abbreviation |
|---|---|---|
| 1 | UCLA Posttraumatic Stress Disorder Reaction Index | UCLA PTSD Index |
| 2 | Child PTSD Symptoms Scale | CPSS |
| 3 | Clinician-Administered PTSD Scale/ Clinician Administered PTSD Scale–Child and Adolescent Version | CAPS/ CAPS-CA |
| 4 | Impact of Events Scale/ The Children's Revised Impact of Events Scale | IES/ CRIES |
| 5 | Parent report of post-traumatic symptoms/Child report of post-traumatic symptoms | PROPS/CROPS |
| 6 | Kiddie-Schedule for Affective Disorders and Schizophrenia | K-SADS |
| 7 | Trauma Symptom Checklist for Children | TSCC |
| 8 | Posttraumatic Cognitions Inventory/Child post-traumatic cognitions inventory | PTCI/CPTCI |
| 9 | Harvard Trauma Questionnaire | HTQ |
| 10 | Post traumatic Stress Scale | PSS |
| 11 | Child Post-Traumatic Stress-Reaction Index | CPTS-RI |
| 12 | The Preschool Age Psychiatric Assessment | PAPA |
| 13 | Anxiety Disorders Interview Schedule | ADIS |



For peer review only - http://bmjopen.bmj.com/site/about/guidelines.xhtml

**PRISMA-P (Preferred Reporting Items for Systematic review and Meta-Analysis Protocols) 2015 checklist: recommended items to address in a systematic review protocol***

| Section and topic | Item No | Checklist item | Reported on Page # |
|---|---|---|---|
| **ADMINISTRATIVE INFORMATION** | | | |
| Title: | | | |
| Identification | 1a | Comparative Efficacy and Acceptability of Psychotherapies for Post-traumatic Stress Disorder in Children and Adolescents: Study Protocol for a Systematic Review and Network Meta-Analysis | 1 |
| Update | 1b | None | |
| Registration | 2 | PROSPERO CRD42016051786 | 3 |
| Authors: | | | |
| Contact | 3a | Yuqing Zhang, Lining Yang, Shuai Yuan, Xiaofeng Jiang, Teng Teng, Peng Xie (Department of Neurology, The First Affiliated Hospital of Chongqing Medical University, Chongqing, China; Institute of Neuroscience and the Collaborative Innovation Center for Brain Science, Chongqing Medical University, Chongqing, China)<br>Xinyu Zhou (Department of Psychiatry, The First Affiliated Hospital of Chongqing Medical University, Chongqing, China)<br>Sarah E. Hetrick ( Orygen, The National Centre of Excellence in Youth Mental Health, and the Centre of Youth Mental Health, University of Melbourne, Melbourne, Australia)<br>John R. Weisz (Department of Psychology, Harvard University, Cambridge, MA, USA)<br>Pim Cuijpers (Department of Clinical, Neuro and Developmental Psychology, Amsterdam Public Health research institute, VU University Amsterdam, Amsterdam, The Netherlands)<br>Jürgen Barth (Institute for Complementary and Integrative Medicine, University Hospital and University of Zurich, Zurich, Switzerland)<br>Cinzia Del Giovane (Department of Diagnostic, Clinical and Public Health Medicine, University of Modena and Reggio Emilia, Modena, Italy)<br>David Cohen (Department of Child and Adolescent Psychiatry, Hôpital Pitié–Salpétrière, Institut des Systèmes Intelligents et Robotiques, Université Pierre et Marie Curie, Paris, France)<br>Donna Gillies (Western Sydney Local Health District - Mental Health, Westmead, Australia)<br><br>Corresponding author :Peng Xie, Department of Neurology, The First Affiliated Hospital of Chongqing Medical University, 1 Youyi Road, Yuzhong District, Chongqing 400016, China; E-mail: xiepeng973@126.com | 1 |
| Contributions | 3b | PX, YZ and XZ conceived the study and drafted the manuscript. SEH, JRW, PC, JB, CDG, DC, DG assisted in protocol design and revision. YZ, XZ, LY, SY, XJ, and TT participated in the search strategy development, and will carry out the most work of study | 15 |

For peer review only - http://bmjopen.bmj.com/site/about/guidelines.xhtml

| | | | |
|---|---|---|---|
| | | selection, risk of bias assessment, and data collection. PX, and SEH will help to revolve the disagreements and check the data. CDG, YZ and XZ participated in the design of data synthesis and analysis, and will conduct the statistical analyses. All the authors have approved the publication of the protocol. | |
| Amendments | 4 | None | |
| Support: | | | |
| Sources | 5a | None | |
| Sponsor | 5b | This work is supported by National Key Research and Development Programm of China (Grant No.2017YFA0505700). | 15 |
| Role of sponsor or funder | 5c | The funders had no role in the protocol design; the writing of the protocol; or the decision to submit the protocol for publication. | 15 |
| **INTRODUCTION** | | | |
| Rationale | 6 | Trauma is common in children and adolescents. More than two thirds of young people have suffered at least one traumatic event, with approximately 15.9% of whom go on to develop post-traumatic stress disorder (PTSD). If children and adolescents do not receive adequate intervention, the symptoms of PTSD may be long-lasting, and have deleterious effects on well-being later in life. Several forms of interventions have been applied to address PTSD symptoms in children and adolescents, from which psychotherapy is the mainstay of treatment. Since the 1990s, psychotherapies have been increasingly applied in the treatment of PTSD in children and adolescents, and the evidence on their efficacy has been reported encouraging in recent reviews. Many types of psychotherapies are now available, such as cognitive behavioral therapy (CBT), eye movement desensitization and reprocessing (EMDR), behavioral therapy (BT), cognitive therapy, psychodynamic therapy and play therapy. Among these, trauma-focused CBT (TF-CBT), a CBT program that utilises cognitive-behavioral techniques with a trauma-focused component, is the most commonly practiced psychotherapy for children and adolescents with PTSD. TF-CBT has been recommended as a first-line treatment by clinical guidelines for PTSD in children and adolescents. However, trauma-focused therapy can be difficult for some therapists to implement, and there are some safety concerns in clinical practice (e.g. symptoms worsening and ending treatment prematurely). Two recent trials that directly compared TF-CBT with non-TF-CBT reported that both of the two treatment modalities were efficacious. Thus, whether the inclusion of a trauma focus component is essential in CBT for PTSD in children and adolescents is still uncertain. Besides, other therapies, such as EMDR and BT, have also been reported effective in treating PTSD symptoms in children and adolescents. However, due to the limited number of randomised controlled trials (RCTs) directly comparing different types of psychotherapies, conventional meta-analyses cannot provide a clear answer regarding the best choice for initial treatment, nor with regard to a hierarchy of these psychotherapies. | 5-6 |
| Objectives | 7 | The aim of this paper is to describe the protocol of a Bayesian network meta-analysis that synthesise all the evidence on psychotherapies for PTSD in children and adolescents and provide clinicians with a reliable treatment hierarchy | 6 |
| **METHODS** | | | |
| Eligibility criteria | 8 | **Types of studies** All randomised controlled trials (RCTs), including cluster-randomised trials and cross-over trials, will be included. However, only the results from the first randomisation period will be considered in the cross-over trials. For the purpose of reducing heterogeneity between trials, we will exclude quasi-randomised trials (e.g., allocation based on the last number of the date of birth) and trials in | 7-8 |

For peer review only - http://bmjopen.bmj.com/site/about/guidelines.xhtml

| | | | |
|---|---|---|---|
| | | which the sample size is less than 10 per study. In addition, to decrease the heterogeneity and inconsistency of this network, trials in which the number of sessions is less than four will be excluded.<br><br>**Types of participants**<br>Studies that enrolled children and adolescents, aged 18 years old or less when they were initially enrolled, will be included in this review. We will apply the following broad criteria to identify the participants: (1) Full PTSD, as diagnosed according to standardised diagnostic interviews based on international classifications (the Diagnostic and Statistical Manual of Mental Disorders (DSM),3,35-38 the International Classification of Diseases (ICD)) or validated scales for PTSD based on DSM/ ICD criteria; (2) Subclinical/Partial PTSD, defined as patients who have experienced psychological trauma and report some subsequent PTSD symptoms in at least one of the four symptom clusters according to DSM-5 (i.e., reexperiencing, avoidance, hyperarousal, negative alterations in cognition and mood); (3) Clinically significant posttraumatic stress symptoms, defined as scoring above a validated cutoff on a PTSD rating scale, such as Kiddie Schedule for Affective Disorders and Schizophrenia (K-SADS), Child PTSD Symptoms Scale (CPSS) and the Impact of Event Scale (IES). We will include trials in which participants have a secondary diagnosis of comorbid general psychiatric disorders, e.g., depressive disorder, anxiety disorder, attention deficit hyperactivity disorder (ADHD) and oppositional defiant disorder (ODD). However, trials in which participants have a diagnosis of acute stress disorder (ASD) or adjustment disorder (AD) will be excluded. Studies where both adults and children/adolescents are included will be eligible for inclusion, if the data for the latter can be obtained separately. Trials conducted in any treatment settings, including outpatient clinics, inpatient services, community clinics and schools will be included.<br><br>**Types of interventions**<br>All RCTs comparing any structured psychotherapy against another structured psychotherapy or any control condition for children and adolescents with PTSD will be included. We will view each of: trauma-focused CBT (TF-CBT), non-trauma-focused CBT (Non-TF-CBT), eye movement desensitization and reprocessing (EMDR), behavioral therapy (BT), cognitive therapy (CT), psychodynamic therapy (DYN), play therapy (PT), and any other structured psychotherapy as independent notes in this network meta-analysis. Trials comparing the same type of psychotherapies, but at different delivery conditions (with or without family involvement), different delivery formats (group, individual, or group plus individual) and different delivery mediums (face-to-face, internet-based) will be considered as the same node in this network meta-analysis. Nevertheless, because TF-CBT is the most commonly studied psychotherapy and recommended by clinical guidelines as first-line choice for children and adolescents with PTSD, if data available, we will separate TF-CBT with different delivery conditions, formats, and mediums as independent nodes.<br>Control conditions can include supportive therapy (ST), waitlist (WL), non-treatment (NT), and treatment as usual (TAU). We will view each of these control conditions as independent nodes in this network meta-analysis.<br>Studies where psychotherapy is used as a combination strategy (e.g., combining psychotherapy with medication) will be excluded, because such designs make it impossible for us to detect effect size of each specific treatment approach. | |
| Information sources | 9 | We will identify relevant trials from systematic searches in the following electronic databases: PubMed, Cochrane, EMBASE, Web of Science, PsycINFO, CINAHL, PILOTS and ProQuest Dissertations. In addition, international trials registers, such as World Health Organization's trials portal, ClinicalTrials.gov and Australian New Zealand Clinical Trials Registry (ANZCTR) will | 10-11 |

For peer review only

| | | | |
|---|---|---|---|
| | | be searched for ongoing trials. Furthermore, we will hand-search relevant key psychiatric and medical journals (including *Behavior Therapy, Child Abuse and Neglect, Child Maltreatment, Journal of Counseling and Development, Counseling Outcome Research and Evaluation, Journal of Anxiety Disorders, Journal of Mental Health Counseling, Journal of Trauma Practice, Journal of Traumatic Stress, Journal of the American Academy of Child and Adolescent Psychiatry*). The reference lists of included trials and reviews identified from initial searches will be scanned for more relevant studies. | |
| Search strategy | 10 | The following search strategy with filters of clinical study will be applied: Condition = [(post-traumatic* or PTSD or PTSS or posttrauma* or trauma* or peritrauma* or peri-trauma* or psychotraumatology or "stress disorder*" or "stress reactions" or "sexually abus*" or "sexual abus*" or "sexually assault*" or "sexual assault*" or "physical abuse*" or "physically abuse*" or "child abuse" or "children abuse" or "natural disaster" or refugee or war or violence or maltreat* or mistreat or acciden* or tsunami* or hurricane* or earthquake or tornado)] AND Intervention = ("trauma focused" or trauma-specific or cogniti* or behavio* or CBT or "eye movement desensitization and reprocessing" or EMDR or EMD or "stress management" or "stress inoculation training" or mindfulness or "art therapy" or "play therapy" or hypnotherapy or biofeedback or counsel* or supportive or interpersonal or bibliotherapy or narrative or narration or exposure* or psychoeducation or "family treatment" or "family therapy" or meditation or relaxation or "problem solving" or "school-based intervention" or "school-based treatment" or "mind-body skills" or "seeking safety" or "mental health" or "writing for recovery" or "psychosocial intervention" or "child centered" or psychodynamic or psychodrama or desensitization or psychological or psychotherap*) AND Age = (adolesc* or boy* or girl* or child* or infant* or juvenil* or minors or pediatri* or paediatri* or pubescen* or puberty or student* or teen* or young or youth* or school* or high-school or preschool* or pre-school* or class*). The following Medical Subject Headings (MeSH) will also be searched: "Stress Disorders" AND "Psychotherapy" AND ("child" OR" adolescent"). | 10-11 |
| Study records: | | | |
| Data management | 11a | We will first manually remove duplicates of initial search results. We will screen citations based on the inclusion and exclusion criteria. | 11-12 |
| Selection process | 11b | Two reviewers (YZ and LY) will independently identify potentially eligible studies from the titles and abstracts of records from the search strategies. Studies will be excluded if both reviewers consider that it does not meet eligibility criteria. Then, the full texts of these potentially eligible studies will be reviewed by the same criteria. The inter-rater reliability of the two reviewers will be calculated to detect their consistency. All disagreements will be discussed and resolved by a senior review author (PX or SEH). The references of relevant reviews and included trials will be checked by YZ and LY. Where multiple publications derive from a common data set, we will select the trials in which the relevant outcomes we predefined in this protocol were reported completely. We will report the reasons for exclusion for each trial in the characteristics of excluded studies list. Finally, a flow chart will be used to present the process of trial screening in this meta-analysis. | 11-12 |
| Data collection process | 11c | A standardised data extraction form will be used by two independent reviewers (YZ and XZ) to record the relevant parameters from the original paper. The reliability of data extraction from the reviewers will also be assessed. Any disagreements will be resolved by a senior review author (PX or SEH). | 12 |
| Data items | 12 | Study characteristics (e.g., title, first author, publication year, publication type, publication journal, location, and sponsor), patient characteristics (e.g., diagnostic criteria for PTSD, type of trauma, severity of PTSD symptoms, comorbidities, and the number, mean age and gender of participants), intervention details (e.g., type of psychotherapy, treatment format, treatment setting, treatment duration, the number of sessions, follow-up duration, and co-interventions) and outcome measures (mean scores, number | 12 |

For peer review only

| | | | |
|---|---|---|---|
| | | of participants, and outcome raters for each pre-defined outcome). A table will be used to present the main characteristics of each study included in this review. | |
| Outcomes and prioritization | 13 | Primary outcome<br>1. Efficacy at post-treatment, as measured using the end point score from PTSD symptom severity rating scales completed by the child, parent, or a clinician. When end point scores are not reported, we will use change scores (if available). Where more than one scale is reported in a trial, we will extract data from the PTSD symptom scales on a hierarchy, which is based on psychometric properties and appropriateness for use with children and adolescents (Table 2). In addition, where a PTSD symptom scale is reported by different raters in a trial, self-rated outcome will be preferred, then the parent or clinician rated outcome, because self-rated outcome tends to result in conservative effect sizes.<br>Secondary outcomes<br>1. Efficacy at follow-up, as measured by the score from PTSD scales at the longest point of follow-up (up to 12 months). The selection priority of PTSD scales will be the same as for efficacy at post-treatment. Data from participants who take part in subsequent treatments (e.g., continuing psychotherapy/pharmacotherapy or booster sessions) will be excluded in the follow-up analysis.<br>2. Acceptability, defined as all-cause discontinuation, as measured by the proportion of patients who discontinued treatment for any cause at the end of treatment. Notably, children and adolescents may discontinue treatment for many different reasons, such as finding it difficult to adhere to long-term treatment, because symptoms worsen, or due to rapid improvement of symptoms.30<br>3. Anxiety symptoms, as measured by the end point score on anxiety symptom severity rating scales. The following scales will be used: Revised Children's Manifest Anxiety Scale (RCMAS), Spence Children's Anxiety Scale (SCAS), Multidimensional Anxiety Scale for Children (MASC), State-Trait Anxiety Inventory for Children (STAIC), Screen for Anxiety and Related Disorders (SCARED). If none of above scales are reported, other valid anxiety scales will be used. When a scale is reported by different raters in a trial, self-rated outcome will be preferred.<br>4. Depressive symptoms, as measured by the end point score on depressive symptom severity rating scales, The following scales will used: Children's Depression Inventory (CDI), Beck depression inventory (BDI), Mood and Feeling Questionnaire (MFQ), Children's Depression Rating Scale Revised (CDRS-R), Hamilton Depression Rating Scale (HAMD). As for anxiety, other valid depression scales will be used if none of the above scales were reported.<br>5. Quality of life and functional improvement (QoL/functioning). If data are available, we will extract continuous outcomes from scales of quality of life and functional improvement measured at post-treatment. The scales of quality of life include Quality of Life Enjoyment and Satisfaction Questionnaire (Q-LES-Q), Pediatric Quality of Life Inventory (QoL Child Report), the Quality of Life Inventory (QoLI), and others. The scales of functional improvement include the Children's Global Assessment Scale (CGAS), the Global Assessment Functioning (GAF), and others. When both of the scales of quality of life and functional improvement are reported in a trial, we will extract the data of quality of life. | 9-10 |
| Risk of bias in individual studies | 14 | Two reviewers (YZ and XZ) will independently assess the methodological quality of the included studies. According to the Cochrane Collaboration's Risk of bias' 2.0 tool, the risk of bias will be rated as "low risk", "high risk", or "some concerns" in the following domains: (1) bias arising from the randomization process, (2) bias due to deviations from intended interventions, (3) bias due to missing outcome data, (4) bias in measurement of the outcome, (5) bias in selection of the reported result, (6) overall bias. The inter-rater reliability of the two reviewers assessing the risk of bias will also be calculated. Any disagreements will be resolved by a senior review author (PX or SEH). | 12 |

For peer review only

| | | | |
|---|---|---|---|
| Data synthesis | 15a | We will perform Bayesian network meta-analysis for each outcome with random-effects model in WinBUGS (version 1.4.3). In addition, as a reference for relative outcomes of network meta-analyses, conventional pairwise meta-analyses will also be performed for each outcome with a random-effects model in Stata (version 13.0). Standardised mean difference (SMD, Cohen's d) will be used as a measure of effect size where outcome is measured on different scales. For dichotomous outcomes, the effect sizes will be calculated as odds ratios (OR). Statistical heterogeneity in each pairwise comparison will be assessed with the $I^2$ statistic and p value. When the mean values or SDs of relative outcomes are missing, we will obtain such value by conversion from p-values, t-values, confidence intervals or standard errors. Missing continuous outcome data will be analysed using the completer data. | 12-13 |
| | 15b | The pooled estimates of network meta-analysis will be obtained using the Markov Chains Monte Carlo method. Two Markov chains will be run simultaneously with different arbitrarily chosen initial values. Trace plots and the Brooks-Gelman-Rubin statistic will be monitored to ensure convergence. When the model is adequately convergent, the foregoing samples will be discarded. Then 100,000 subsequent simulations will be run as the posterior summaries. All results will be reported as effect sizes (SMD or OR) and their 95% credible intervals (CrI). We will assume a common heterogeneity parameter for each comparison and assess the global heterogeneity using the $I^2$ statistic with 'gemtc' package in R (version 3.2.2). Furthermore, the inconsistency between direct and indirect evidence will be evaluated, which will include the assessment of global inconsistency (by comparing the fit and parsimony of consistency and inconsistency models), local inconsistency (by calculating the difference among direct and indirect evidence in closed loops in the network) and the inconsistency calculated by node splitting method (by assessing the difference between direct and indirect estimates within a particular comparison). Probability values will be summarised and reported as surface under the cumulative ranking curve (SUCRA) to provide a hierarchy of the treatments. | 13 |
| | 15c | Subgroup analysis<br>Where possible, we will conduct network meta-regression of data on primary outcome, to evaluate the influence of the following potential moderators: (1) age group; (2) sex ratio; (3) number of sessions; (4) sample size; (5) risk of bias; (6) trauma types (acute/single trauma vs chronic trauma); (7) diagnosis criteria (youth with a diagnosis vs with subsyndromal symptoms); (8) source of outcome measure (self-rated vs observer-rated). If the data are insufficient to conduct subgroup analyses for some moderators, we will perform sensitivity analyses by omitting specific trials from the overall analysis. | 13-14 |
| Meta-bias(es) | 16 | Comparison-adjusted funnel plots and Egger's test will be used for each outcome to examine whether there is dominant publication bias exist in this network meta-analysis. | 14 |
| Confidence in cumulative evidence | 17 | We will evaluate the quality of evidence for primary outcome by using the GRADE framework, which characterises the quality of a body of evidence on the basis of the study limitations, imprecision, inconsistency, indirectness, and publication bias for network estimates. | 14 |

*** It is strongly recommended that this checklist be read in conjunction with the PRISMA-P Explanation and Elaboration (cite when available) for important clarification on the items. Amendments to a review protocol should be tracked and dated. The copyright for PRISMA-P (including checklist) is held by the PRISMA-P Group and is distributed under a Creative Commons Attribution Licence 4.0.**

*From: Shamseer L, Moher D, Clarke M, Ghersi D, Liberati A, Petticrew M, Shekelle P, Stewart L, PRISMA-P Group. Preferred reporting items for systematic review and meta-analysis protocols (PRISMA-P) 2015: elaboration and explanation. BMJ. 2015 Jan 2;349(jan02 1):g7647.*

For peer review only

BMJ Open

# Comparative Efficacy and Acceptability of Psychotherapies for Post-traumatic Stress Disorder in Children and Adolescents: Study Protocol for a Systematic Review and Network Meta-Analysis

| | |
|---|---|
| Journal: | *BMJ Open* |
| Manuscript ID | bmjopen-2017-020198.R1 |
| Article Type: | Protocol |
| Date Submitted by the Author: | 22-Dec-2017 |
| Complete List of Authors: | Zhang, Yuqing; The First Affiliated Hospital of Chongqing Medical University, Department of Neurology<br>Zhou, Xinyu; The First Affiliated Hospital of Chongqing Medical University, Department of Psychiatry<br>Yang, Lining; The First Affiliated Hospital of Chongqing Medical University, Department of Neurology<br>Hetrick, Sarah; University of Melbourne, Centre for Youth Mental Health; University of Auckland, Department of Psychological Medicine<br>Weisz, John; Harvard University, Department of Psychology<br>Cuijpers, Pim; VU University Amsterdam, Department of Clinical Psychology<br>Barth, Juergen; University Hospital and University of Zurich, Institute for Complementary and Integrative Medicine<br>Del Giovane, Cinzia; University of Bern, Institute of Primary Health Care (BIHAM)<br>Yuan, Shuai; The First Affiliated Hospital of Chongqing Medical University, Department of Neurology<br>Cohen, David; Institut des Systèmes Intelligents et de Robotiques, UPMC Univ Paris 06, Department of Child and Adolescent Psychiatry<br>Gillies, Donna; Western Sydney Local Health District, Mental Health<br>Jiang, Xiaofeng; The First Affiliated Hospital of Chongqing Medical University, Department of Neurology<br>Teng, Teng; The First Affiliated Hospital of Chongqing Medical University, Department of Neurology<br>Xie, Peng; The First Affiliated Hospital of Chongqing Medical University, Department of Neurology; Chongqing Medical University, Institute of Neuroscience and the Collaborative Innovation Center for Brain Science |
| <b>Primary Subject Heading</b>: | Mental health |
| Secondary Subject Heading: | Evidence based practice, Pharmacology and therapeutics, Paediatrics |
| Keywords: | post-traumatic stress disorder, Child & adolescent psychiatry < PSYCHIATRY, psychotherapy, network meta-analysis, systematic review |
| | |

For peer review only

# Comparative Efficacy and Acceptability of Psychotherapies for Post-traumatic Stress Disorder in Children and Adolescents: Study Protocol for a Systematic Review and Network Meta-Analysis

Yuqing Zhang,[1] Xinyu Zhou,[2] Lining Yang,[1] Sarah E. Hetrick,[3] John R. Weisz,[4] Pim Cuijpers,[5] Jürgen Barth,[6] Cinzia Del Giovane,[7] Shuai Yuan,[1] David Cohen,[8] Donna Gillies,[9] Xiaofeng Jiang,[1] Teng Teng,[1] Peng Xie[1]

[1] Department of Neurology, The First Affiliated Hospital of Chongqing Medical University, Chongqing, China; Institute of Neuroscience and the Collaborative Innovation Center for Brain Science, Chongqing Medical University, Chongqing, China

[2] Department of Psychiatry, The First Affiliated Hospital of Chongqing Medical University, Chongqing, China

[3] Department of Psychological Medicine, University of Auckland, and the Centre of Youth Mental Health, University of Melbourne, Melbourne, Australia

[4] Department of Psychology, Harvard University, Cambridge, MA, USA

[5] Department of Clinical, Neuro and Developmental Psychology, Amsterdam Public Health research institute, Vrije Universiteit Amsterdam, The Netherlands

[6] Institute for Complementary and Integrative Medicine, University Hospital and University of Zurich, Zurich, Switzerland

[7] Institute of Primary Health Care (BIHAM), University of Bern, Switzerland

[8] Department of Child and Adolescent Psychiatry, Hôpital Pitié–Salpétrière, Institut des Systèmes Intelligents et Robotiques, Université Pierre et Marie Curie, Paris, France

[9] Western Sydney Local Health District - Mental Health, Westmead, Australia

YZ, XZ and LY contributed equally to this work.

**Direct correspondence to:**

Prof Peng Xie, Department of Neurology, The First Affiliated Hospital of Chongqing Medical University, Yuzhong District, Chongqing, China; E-mail: xiepeng973@126.com.

Keywords: post-traumatic stress disorder, child, adolescent, psychotherapy, network meta-analysis, systematic review



For peer review only - http://bmjopen.bmj.com/site/about/guidelines.xhtml

Word count: 3333.

For peer review only



For peer review only - http://bmjopen.bmj.com/site/about/guidelines.xhtml

**ABSTRACT**

**Introduction**: Post-traumatic stress disorder (PTSD) is common among children and adolescents who are exposed to trauma, and it is often associated with significant negative impacts on their psychosocial functioning and quality of life. Many types of psychotherapies have been found to be effective for PTSD in children and adolescents. However, due to the lack of direct comparisons between different psychotherapies, the hierarchy of treatment efficacy is still unclear. Therefore, we plan to conduct a systematic review and network meta-analysis to evaluate the efficacy and acceptability of various types of psychotherapies for PTSD in children and adolescents.

**Methods and analysis**: A systematic search will be conducted among eight electronic databases, including PubMed, Cochrane, EMBASE, Web of Science, PsycINFO, CINAHL, PILOTS and ProQuest Dissertations, from inception to October 2017. Randomised controlled trials (RCTs), regardless of language, publication year, and publication type, comparing any psychotherapies for PTSD to any control condition or alternative treatment in children and adolescents (18 years old or less) diagnosed with full or subclinical PTSD will be included. Study duration and the number of treatment sessions will not be limited. The primary outcome will be PTSD symptom severity at post-treatment as measured by a rating scale reported by the child, parent, or a clinician. The secondary outcomes will include (1) efficacy at follow-up; (2) acceptability (all-cause discontinuation); (3) anxiety symptom severity; (4) depressive symptom severity; and (5) quality of life and functional improvement. Bayesian network meta-analyses for all relative outcome measures will be performed. We will conduct subgroup and sensitivity network meta-analyses to determine whether the findings are affected by study characteristics. The quality of the evidence contributing to network estimates of the primary outcome will be evaluated by the GRADE framework.

**Ethics and dissemination:** No ethical issues are foreseen. The results will be published in a peer-reviewed journal, which will be disseminated electronically and in print. This network meta-analysis may be updated to inform and guide the clinical management of PTSD in children and adolescents.



For peer review only - http://bmjopen.bmj.com/site/about/guidelines.xhtml

**Trial registration number:** PROSPERO CRD42016051786.

For peer review only



For peer review only - http://bmjopen.bmj.com/site/about/guidelines.xhtml

**Strengths and limitations of this study**

1. Bayesian network meta-analysis can simultaneously compare various types of treatments by integrating all the best evidence (direct and indirect evidence) to estimate the interrelations across all treatments and establish a treatment hierarchy for psychotherapies for post-traumatic stress disorder in children and adolescents.

2. A number of outcomes will be used to comprehensively assess efficacy at post-treatment and follow-up, acceptability, anxiety and depressive symptom severity, quality of life and functional improvement.

3. This study will help to guide clinical decision-making regarding the relative efficacy of various types of psychotherapy, and various delivery modalities, in the treatment of post-traumatic stress disorder in children and adolescents.

4. Subgroup and sensitivity network meta-analyses will help to find potential moderators that affect the efficacy of psychotherapies.

5. The limitations of included studies will be assessed using the Cochrane Collaboration's Risk of bias' 2.0 tool and the quality of evidence for network estimates of the primary outcome will be evaluated by the GRADE framework.

For peer review only



For peer review only - http://bmjopen.bmj.com/site/about/guidelines.xhtml

## BACKGROUND

Many children and adolescents are exposed to trauma. ,with more than two thirds reporting at least 1 traumatic event by 16 years of age.[1] Post-traumatic stress disorder (PTSD) is one of the most common mental disorders among children and adolescents who have experienced trauma, with an overall prevalence of 15.9%.[2] As described in the Diagnostic and Statistical Manual of Mental Disorders, 5th edition (DSM-5), PTSD diagnostic criteria includes four symptom clusters: (1) re-experiencing of the traumatic event, (2) avoidance of stimuli associated with the traumatic event, (3) negative alterations in cognition and mood, (4) hyperarousal.[3] Trauma exposure is an essential factor in order to be able to diagnose PTSD, which can include physical or sexual abuse, war or terrorism, natural or man-made disasters, witnessing domestic violence, catastrophic illnesses, vehicle or other accidents.[4,5] PTSD is correlated with various adverse consequences for children and adolescents in cognitive, emotional, social, academic, and other functional domains.[6] In addition, PTSD commonly co-occurs with other psychiatric conditions, such as major depressive disorder,[7] anxiety disorder,[7] substance abuse,[8] and ADHD.[9] PTSD diagnosis is accompanied by a significant reduction in quality of life, and it is estimated that successful treatment could save 2.05 quality adjusted life years (QALYs) per child or adolescent with PTSD.[10]

Several forms of interventions have been applied to address PTSD symptoms in children and adolescents, from which psychotherapy is the mainstay of treatment.[11] Although some medications (e.g., fluoxetine, paroxetine and venlafaxine) have shown a significant but small superiority over placebo (mean effect size=0.23) in PTSD treatment for adults,[12] their use in children and adolescents is still limited because (1) the evidence of the effectiveness of pharmacological treatments for children and adolescents with PTSD is scant;[13] and, (2) medications have been associated with safety concerns in children and adolescents, including evidence of increased risk of suicidality in children and adolescents treated with antidepressants.[14,15] Therefore, clinical guidelines recommend that psychotherapy should be used for the initial treatment of PTSD in children and adolescents.[16]



For peer review only - http://bmjopen.bmj.com/site/about/guidelines.xhtml

Since the 1990s, psychotherapies have been increasingly applied in the treatment of PTSD in children and adolescents, and recent reviews have reported encouraging results on their efficacy.[17-19] Evidence from Cochrane reviews found that psychological therapies are not only effective in preventing PTSD in children and adolescents who have undergone trauma, but also effective in treating PTSD in children and adolescents.[17,18] Many types of psychotherapies are now available, such as cognitive behavioral therapy (CBT), eye movement desensitization and reprocessing (EMDR), behavioral therapy (BT), cognitive therapy, psychodynamic therapy and play therapy. Among these, trauma-focused CBT (TF-CBT), a CBT program that uses cognitive-behavioral techniques with a trauma-focused component, is the most commonly practiced psychotherapy for children and adolescents with PTSD.[20] TF-CBT has been recommended as a first-line treatment by clinical guidelines for PTSD in children and adolescents.[16,21] However, trauma-focused therapy can be difficult for some therapists to implement,[22,23] and there are some safety concerns in clinical practice (e.g. symptoms worsening and patients ending treatment prematurely).[24,25] Two recent trials that directly compared TF-CBT with non-TF-CBT alternatives reported that both were efficacious.[23,26] Thus, whether the inclusion of a trauma-focused component is essential in CBT for PTSD in children and adolescents is still uncertain. Moreover, other therapies, such as EMDR and BT, have also been reported effective in treating PTSD symptoms in children and adolescents.[27,28] However, due to methodological limitations, conventional meta-analyses cannot simultaneously compare all these treatments. Therefore, they cannot provide a clear answer regarding the best choice for initial treatment, nor can they provide a hierarchy of these psychotherapies.

Network meta-analysis is a newly developed method for evidence synthesis, which is able to integrate direct evidence (from studies directly comparing interventions) with indirect evidence (derived from separate studies addressing a common reference condition) from multiple treatment comparisons to estimate the interrelations across all treatments.[29] This approach enables a simultaneous comparison of multiple treatments, and can provide hierarchical evidence to guide clinical practice. Using this method, our group has produced meta-analyses comparing the effects of



For peer review only - http://bmjopen.bmj.com/site/about/guidelines.xhtml

different psychotherapies for the treatment of anxiety disorder[30] and depressive disorder[31] in children and adolescents. In the present study we describe the methods to undertake a network meta-analysis to complement those previous reports that will focus on the efficacy of psychotherapies for children and adolescents with PTSD. By performing a well-designed Bayesian network meta-analysis, we aim to provide a higher level of evidence that can inform clinical guidelines for PTSD treatment in young people.

## METHODS

### Criteria for included studies

#### *Types of studies*

All randomised controlled trials (RCTs), including cluster-randomised trials and cross-over trials, will be included. However, only the results from the first randomisation period will be considered in the cross-over trials. For the purpose of reducing heterogeneity between trials, we will exclude quasi-randomised trials (e.g., allocation based on the last number of the date of birth) and trials in which the sample size is less than 10 per study. Study duration and the number of treatment sessions will not be limited.

#### *Types of participants*

Studies that enrolled children and adolescents, aged 18 years old or less when they were initially enrolled, will be included in this review. Given that children with significant PTSD symptoms who do not meet full criteria for a PTSD diagnosis often have comparable functional impairment to those with a PTSD diagnosis,[32-34] the clinical guideline suggested that treatment decisions should be based on symptom severity and functional impairment, rather than whether or not they have an actual PTSD diagnosis.[16] Therefore, we will apply the following broad criteria to identify the participants: (1) Full PTSD, as diagnosed according to standardised diagnostic interviews based on international classifications (the Diagnostic and Statistical Manual of Mental Disorders (DSM),[3,35-38] the International Classification of Diseases (ICD) [39,40]) or validated scales for PTSD based on DSM/ ICD criteria;[41-44] (2) Subclinical/Partial PTSD, defined



For peer review only - http://bmjopen.bmj.com/site/about/guidelines.xhtml

as patients who have experienced psychological trauma and report some subsequent PTSD symptoms in at least one of the four symptom clusters according to DSM-5 (i.e., reexperiencing, avoidance, hyperarousal, negative alterations in cognition and mood);[45] (3) Clinically significant posttraumatic stress symptoms, defined as scoring above a validated cutoff on a PTSD rating scale, such as the Kiddie Schedule for Affective Disorders and Schizophrenia (K-SADS)[42], Child PTSD Symptoms Scale (CPSS)[46] and the Impact of Event Scale (IES)[47]. We will include trials in which participants have a secondary diagnosis of comorbid general psychiatric disorders, e.g., depressive disorder, anxiety disorder, attention deficit hyperactivity disorder (ADHD) and oppositional defiant disorder (ODD). However, trials in which participants have a diagnosis of acute stress disorder (ASD) or adjustment disorder (AD) will be excluded. Studies where both adults and children/adolescents are included will be eligible for inclusion, if the data for the latter can be obtained separately. Studies where it is not clear what happened to the patients who withdrew from the study will not be excluded, and all these patients will be counted as all-cause discontinuation. Trials conducted in any treatment settings, including outpatient clinics, inpatient services, community clinics and schools will be included.

***Types of interventions***

All RCTs comparing any psychotherapy against another psychotherapy or any control condition for children and adolescents with PTSD will be included. We will view each of: trauma-focused CBT (TF-CBT), non-trauma-focused CBT (Non-TF-CBT), eye movement desensitization and reprocessing (EMDR), behavioral therapy (BT), cognitive therapy (CT), psychodynamic therapy (DYN), play therapy (PT), and any other psychotherapy as independent nodes in this network meta-analysis. Trials comparing the same type of psychotherapies, but at different delivery conditions (with or without family involvement), different delivery formats (group, individual, or group plus individual) and different delivery mediums (face-to-face, internet-based) will be considered as the same node in this network meta-analysis. Nevertheless, because TF-CBT is the most commonly studied psychotherapy and recommended by clinical guidelines as first-line choice for children and adolescents with PTSD,[16,21] if data available, we will separate TF-CBT with different



For peer review only - http://bmjopen.bmj.com/site/about/guidelines.xhtml

delivery conditions, formats, and mediums as independent nodes.

Control conditions can include waitlist (WL), non-treatment (NT), and treatment as usual (TAU). We will view each of these control conditions as independent nodes in this network meta-analysis. The detailed description of each treatment and control condition are presented in Table 1. According to the principles described in this table, two reviewers will independently perform the classification of all conditions in each trial. Any disagreements will be discussed among the review team, and any unclear information will be requested from the relevant authors.

Studies where psychotherapy is used as a combination strategy (e.g., combining psychotherapy with medication) will be excluded, because such designs make it impossible for us to estimate the effect size of each specific treatment approach. We will not exclude studies that enrolled patients who had used medications in the past, provided that their medication status was not changed for at least one month prior to study entry and for the study period.

***Types of outcome measures***

*Primary outcome*

1. Efficacy at post-treatment, as measured using the end point score from PTSD symptom severity rating scales completed by the child, parent, or a clinician.[48] When end point scores are not reported, we will use change scores (if available).[49] Where more than one scale is reported in a trial, we will extract data from the PTSD symptom scales on a hierarchy, which is based on psychometric properties and appropriateness for use with children and adolescents (Table 2). In addition, where a PTSD symptom scale is reported by different raters in a trial, self-rated outcome will be preferred, then the parent or clinician rated outcome, because self-rated outcome tends to result in more conservative effect sizes.[50] Where dichotomous efficacy outcomes, instead of continuous scores, are reported in a trial, we will contact the relevant authors to request the data we need. If they don’t respond the data will not be used.

*Secondary outcomes*

1. Efficacy at follow-up, as measured by the score from PTSD scales at the longest point of follow-up (up to 12 months). The selection priority of PTSD scales will be the same as for efficacy at post-treatment. Data from participants



For peer review only - http://bmjopen.bmj.com/site/about/guidelines.xhtml

who take part in subsequent treatments (e.g., continuing psychotherapy/pharmacotherapy or booster sessions) will be excluded in the follow-up analysis.

2. Acceptability, defined as all-cause discontinuation, as measured by the proportion of patients who discontinued treatment for any cause at the end of treatment. Notably, children and adolescents may discontinue treatment for many different reasons, such as finding it difficult to adhere to long-term treatment, because symptoms worsen, or due to rapid improvement of symptoms.[30]

3. Anxiety symptoms, as measured by the end point score on anxiety symptom severity rating scales. The following scales will be used: Revised Children's Manifest Anxiety Scale (RCMAS), Spence Children's Anxiety Scale (SCAS), Multidimensional Anxiety Scale for Children (MASC), State-Trait Anxiety Inventory for Children (STAIC), Screen for Anxiety and Related Disorders (SCARED). If none of above scales are reported, other valid anxiety scales will be used. When a scale is reported by different raters in a trial, self-rated outcome will be preferred.

4. Depressive symptoms, as measured by the end point score on depressive symptom severity rating scales. The following scales will used: Children's Depression Inventory (CDI), Beck depression inventory (BDI), Mood and Feeling Questionnaire (MFQ), Children's Depression Rating Scale Revised (CDRS-R), Hamilton Depression Rating Scale (HAMD). As for anxiety, other valid depression scales will be used if none of the above scales were reported.

5. Quality of life and functional improvement (QoL/functioning). If data are available, we will extract continuous outcomes from scales of quality of life and functional improvement measured at post-treatment. The scales of quality of life include Quality of Life Enjoyment and Satisfaction Questionnaire (Q-LES-Q), Pediatric Quality of Life Inventory (QoL Child Report), the Quality of Life Inventory (QoLI), and others. The scales of functional improvement include the Children's Global Assessment Scale (CGAS), the Global Assessment Functioning (GAF), and others. When scales of quality of life and functional improvement are both reported in a trial, we will extract the data of quality of life.

***Search strategy***



For peer review only - http://bmjopen.bmj.com/site/about/guidelines.xhtml

For peer review only

We will identify relevant trials from systematic searches in the following electronic databases: PubMed, Cochrane, EMBASE, Web of Science, PsycINFO, CINAHL, PILOTS and ProQuest Dissertations. The timeline will be from database inception to October 2017. The full search strategy for each database is included in the supplementary material. In addition, international trials registers, such as World Health Organization's trials portal, ClinicalTrials.gov and Australian New Zealand Clinical Trials Registry (ANZCTR) will be searched for ongoing trials. Furthermore, we will search the presentations at European Psychiatric Association (EPA), European College of Neuropsychopharmacology (ECNP), American Psychological Association (APA), American Psychiatric Association (APA) and European Society for Child and Adolescent Psychiatry (ESCAP) congresses and hand-search relevant key psychiatric, psychological and medical journals (including *Behavior Therapy, Child Abuse and Neglect, Child Maltreatment, Journal of Counseling and Development, Counseling Outcome Research and Evaluation, Journal of Anxiety Disorders, Journal of Mental Health Counseling, Journal of Trauma Practice, Journal of Traumatic Stress, Journal of the American Academy of Child and Adolescent Psychiatry, Journal of Consulting and Clinical Psychology, Behaviour research and therapy*). The reference lists of included trials and reviews identified from initial searches will be scanned for more relevant studies. All relevant authors will be contacted to complement the incomplete data.

**Study selection and data extraction**

***Selection of trials***

Two reviewers (YZ and LY) will independently identify potentially eligible studies from the titles and abstracts of records from the search strategies. Studies will be excluded if both reviewers consider that it does not meet eligibility criteria. Then, the full texts of these potentially eligible studies will be reviewed by the same criteria. The inter-rater reliability of the two reviewers will be calculated to detect their consistency. All disagreements will be discussed and resolved by a senior review author (PX or SEH). The references of relevant reviews and included trials will be checked by YZ and LY. Where multiple publications derive from a common data set, we will select the trials in which the relevant



For peer review only - http://bmjopen.bmj.com/site/about/guidelines.xhtml

outcomes we predefined in this protocol were reported completely. We will report the reasons for exclusion for each trial in the characteristics of excluded studies list. Finally, a flow chart will be used to present the process of trial screening in this meta-analysis.

***Risk of bias assessment***

Two reviewers (YZ and XZ) will independently assess the methodological quality of the included studies. According to the Cochrane Collaboration's Risk of bias' 2.0 tool,[51] the risk of bias will be rated as "low risk", "high risk", or "some concerns" in the following domains: (1) bias arising from the randomization process, (2) bias due to deviations from intended interventions, (3) bias due to missing outcome data, (4) bias in measurement of the outcome, (5) bias in selection of the reported result, (6) overall bias. The inter-rater reliability of the two reviewers assessing the risk of bias will also be calculated. Any disagreements will be resolved by a senior review author (PX or SEH).

***Data extraction***

A standardised data extraction form will be used by two independent reviewers (YZ and XZ) to record the relevant parameters from the original paper, including study characteristics (e.g., title, first author, publication year, publication type, publication journal, location, and sponsor), patient characteristics (e.g., diagnostic criteria for PTSD, type of trauma, severity of PTSD symptoms, comorbidities, and the number, mean age and gender of participants), intervention details (e.g., type of psychotherapy, treatment format, treatment setting, treatment duration, the number of sessions, follow-up duration, and co-interventions) and outcome measures (mean scores, number of participants, and outcome raters (i.e., self rated or observer rated) for each pre-defined outcome). A table will be used to present the main characteristics of each study included in this review. The reliability of data extraction from the reviewers will also be assessed. Any disagreements will be resolved by a senior review author (PX or SEH).

**Statistical analysis**

We will perform Bayesian network meta-analysis for each outcome with random-effects model in WinBUGS



For peer review only - http://bmjopen.bmj.com/site/about/guidelines.xhtml

(version 1.4.3). In addition, as a reference for relative outcomes of network meta-analyses, conventional pairwise meta-analyses will also be performed for each outcome with a random-effects model in Stata (version 13.0). Standardised mean difference (SMD, Cohen's d) will be used as a measure of effect size where outcome is measured on different scales. For dichotomous outcome (all-cause discontinuation), the effect sizes will be calculated as odds ratios (OR). Statistical heterogeneity in each pairwise comparison will be assessed with the $I^2$ statistic and p value.[52] When the mean values or SDs of continuous outcomes are missing, we will compute values by conversion from p-values, t-values, confidence intervals or standard errors.[53] Missing continuous outcome data will be analysed using completer data.

The pooled estimates of network meta-analysis will be obtained using the Markov Chains Monte Carlo method. Two Markov chains will be run simultaneously with different arbitrarily chosen initial values. Trace plots and the Brooks-Gelman-Rubin statistic will be monitored to ensure convergence.[54] When the model is adequately convergent, the foregoing samples will be discarded. Then 100,000 subsequent simulations will be run as the posterior summaries. All results will be reported as effect sizes (SMD or OR) and their 95% credible intervals (CrI). We will assume a common heterogeneity parameter for each comparison and assess the global heterogeneity using the $I^2$ statistic with 'gemtc' package in R (version 3.2.2). Furthermore, the inconsistency between direct and indirect evidence will be evaluated, which will include the assessment of global inconsistency (by comparing the fit and parsimony of consistency and inconsistency models), local inconsistency (by calculating the difference among direct and indirect evidence in closed loops in the network)[55] and the inconsistency calculated by node splitting method (by assessing the difference between direct and indirect estimates within a particular comparison).[56] Probability values will be summarised and reported as surface under the cumulative ranking curve (SUCRA) to provide a hierarchy of the treatments.[57]

***Subgroup analysis***

Where possible, we will conduct network meta-regression of data on primary outcome, to evaluate the influence of the following potential moderators: (1) age group; (2) sex ratio; (3) number of sessions; (4) sample size; (5) risk of bias;



For peer review only - http://bmjopen.bmj.com/site/about/guidelines.xhtml

(6) trauma types (acute/single trauma vs chronic trauma); (7) diagnosis criteria (youth with a diagnosis vs with subsyndromal symptoms); (8) source of outcome measure (self-rated vs observer-rated). If the data are insufficient to conduct subgroup analyses for some moderators, we will perform sensitivity analyses by omitting specific trials from the overall analysis.

***Other analyses***

Comparison-adjusted funnel plots and Egger's test will be used for each outcome to examine whether there is dominant publication bias exist in this network meta-analysis.[56] In addition, we will evaluate the quality of evidence for primary outcome by using the GRADE framework, which characterises the quality of a body of evidence on the basis of the study limitations, imprecision, inconsistency, indirectness, and publication bias for network estimates.[58]

**Discussion**

This systematic review and network meta-analysis will comprehensively evaluate the comparative efficacy and acceptability of psychological interventions for children and adolescents with PTSD. The results of this research will provide evidence to inform a hierarchy of comparative efficacy at post-treatment, efficacy with regard to PTSD symptoms at follow-up, as well as in terms of acceptability (all-cause discontinuation), improvement of anxiety symptoms, improvement of depressive symptoms, and quality of life and functional improvement. We expect that the findings could assist patients, clinicians and healthcare providers to make a better-informed choice in treatment selection.

There are some limitations in this protocol. First, due to the fact that PTSD commonly co-occurs with other psychiatric comorbidities, we will not exclude trials in which patients with comorbidity were enrolled. This will enhance the generalizability of this study; however, it will also raise the risk of bias for outcomes. Further individual patient data meta-analysis will be helpful to investigate the influence of this factor. Second, some trials may tend to report their favourite outcomes. Although we have predefined the data selection criteria for each outcome, this selection bias could not be completely eliminated.



For peer review only - http://bmjopen.bmj.com/site/about/guidelines.xhtml

**Ethics and dissemination**

This network meta-analysis will use data in the public domain. Therefore, no formal ethical assessment and informed consent are required. The results will be published in a peer-reviewed journal and disseminated electronically and in print.

For peer review only



For peer review only - http://bmjopen.bmj.com/site/about/guidelines.xhtml

**Contributors**

PX, YZ and XZ conceived the study and drafted the manuscript. SEH, JRW, PC, JB, CDG, DC, DG assisted in protocol design and revision. YZ, XZ, LY, SY, XJ, and TT participated in the search strategy development, and will carry out the most work of study selection, risk of bias assessment, and data collection. PX, and SEH will help to revolve the disagreements and check the data. CDG, YZ and XZ participated in the design of data synthesis and analysis, and will conduct the statistical analyses. All the authors have approved the publication of the protocol.

**Funding** This work is supported by National Key Research and Development Programm of China (Grant No.2017YFA0505700). The funders had no role in the protocol design; the writing of the protocol; or the decision to submit the protocol for publication.

**Competing interests** SEH is an editor of the Cochrane Common Mental Disorders Group, and an author on the Cochrane systematic review on treatments for PTSD in young people. During the last two years, David Cohen reported past consultation for or the receipt of honoraria from Otsuka, Shire, Lundbeck and Roche. DG is the primary author of two reviews of psychological therapies for children and adolescents who were diagnosed with PTSD or exposed to trauma. YZ, XZ, LY, JRW, PC, JB, CDG, SY, XJ, TT, and PX declare no competing interests.

**Provenance and peer review** Not commissioned; externally peer-reviewed.

**Open Access** This is an Open Access article distributed in accordance with the Creative Commons Attribution- Non Commercial (CC BY-NC 4.0) license, which permits others to distribute, remix, adapt, build upon this work non-commercially, and license their derivative works on different terms, provided the original work is properly cited and the use is non-commercial. See: http:// creativecommons.org/- licenses/by-nc/4.0/

For peer review only



For peer review only - http://bmjopen.bmj.com/site/about/guidelines.xhtml

1 Copeland WE, Keeler G, Angold A, et al. Traumatic events and post-traumatic stress in childhood. *Arch General Psychiatry* 2007;64:577–84.

2. Alisic E, Zalta AK, Van Wesel F, et al. Rates of post-traumatic stress disorder in trauma-exposed children and adolescents: meta-analysis. *Br J Psychiatry* 2014;204:335-40.

3. American Psychiatric Association. Diagnostic and statistical manual of mental disorders. 5th edn. Arlington, VA: American Psychiatric Publishing, 2013.

4. Donnelly CL. Amaya-Jackson L. Post-traumatic stress disorder in children and adolescents: epidemiology, diagnosis and treatment options. *Paediatr Drugs* 2002;4:159-70.

5. Taylor TL, Chemtob CM. Efficacy of treatment for child and adolescent traumatic stress. *Arch Pediatr Adolesc Med* 2004;158:786-91.

6. Dorsey S, McLaughlin KA, Kerns SEU, et al. Evidence Base Update for Psychosocial Treatments for Children and Adolescents Exposed to Traumatic Events. *J Clin Child Adolesc Psychol* 2017;46:303-30.

7. Dixon A, Howie P, Starling J. Trauma exposure, posttraumatic stress, and psychiatric comorbidity in female juvenile offenders. *J Am Acad Child Adolesc Psychiatry* 2005;44:798-806.

8. Lipschiz D, Rasmusson AM, Anyan W, et al. Posttraumatic stress disorder and substance use in inner city girls. *J Nervous Mental Disease* 2003;191:714-21.

9. Ford JD, Racusin R, Ellis CG, et al. Child maltreatment, other trauma exposure and posttraumatic symptomatology among children with oppositional disorder and attention deficit hyperactivity disorders. *Child Maltreat* 2000;5:205-17.

10. Gospodarevskaya E. Post-traumatic stress disorder and quality of life in sexually abused Australian children. *J Child Sex Abus* 2013;22:277-96.

11. Keeshin BR, Strawn JR. Psychological and pharmacologic treatment of youth with posttraumatic stress disorder: an



For peer review only - http://bmjopen.bmj.com/site/about/guidelines.xhtml

For peer review only

evidence-based review. *Child Adolesc Psychiatr Clin N Am* 2014;23:399-411,

12. Hoskins M, Pearce J, Bethell A, et al. Pharmacotherapy for post-traumatic stress disorder: systematic review and meta-analysis. *Br J Psychiatry* 2015;206:93-100.
13. Bajor LA, Ticlea AN, Osser DN. The Psychopharmacology Algorithm Project at the Harvard South Shore Program: an update on posttraumatic stress disorder. *Harv Rev Psychiatry* 2011;19:240-58.
14. U.S. Food and Drug Administration (FDA). Suicidality in children and adolescents being treated with antidepressant medications. http://www.fda.gov/drugs/drugsafety/postmarketdrugsafetyinformationforpatientsandproviders/ ucm161679.htm (accessed 2 Aug 2017).
15. Cipriani A, Zhou X, Del Giovane C, et al. Comparative efficacy and tolerability of antidepressants for major depressive disorder in children and adolescents: a network meta-analysis. *Lancet* 2016;388:881-90.
16. Cohen JA, Bukstein O, Walter H, et al. Practice Parameter for the assessment and treatment of children and adolescents with posttraumatic stress disorder. *J Am Acad Child Adolesc Psychiatry* 2010;49:414-30.
17. Gillies D, Taylor F, Gray C, et al. Psychological therapies for the treatment of post-traumatic stress disorder in children and adolescents. *Cochrane Database Syst Rev* 2012;12:CD006726.
18. Gillies D, Maiocchi L, Bhandari AP, et al. Psychological therapies for children and adolescents exposed to trauma. *Cochrane Database Syst Rev* 2016;10:CD012371.
19. Gutermann J, Schreiber F, Matulis S, et al. Psychological treatments for symptoms of posttraumatic stress disorder in children, adolescents, and young adults: a meta-analysis. *Clin Child Fam Psychol Rev* 2016;19:77-93.
20. Lenz AS, Hollenbaugh KM. Meta-analysis of trauma-focused cognitive behavioral therapy for treating PTSD and co-occurring depression among children and adolescents. *Counseling Outcome Research and Evaluation* 2015;6:18-32.
21. National Collaborating Centre for Mental Health (NCCMH). Post-traumatic stress disorder: The management of

For peer review only



For peer review only - http://bmjopen.bmj.com/site/about/guidelines.xhtml

PTSD in adults and children in primary and secondary care. National Clinical Practice Guideline Number 26. London: National Institute for Clinical Excellence (NICE), 2005.

22. Cohen J, Mannarino AP, Deblinger E. Treating Trauma and Traumatic Grief in Children and Adolescents. New York: Guilford; 2006.

23. Salloum A, Overstreet S. Grief and trauma intervention for children after disaster: exploring coping skills versus trauma narration. *Behav Res Ther* 2012;50:169-79.

24. Linden M, Schermuly-Haupt ML. Definition, assessment and rate of psychotherapy side effects. *World Psychiatry* 2014;13:306-9.

25. Nutt DJ, Sharpe M. Uncritical positive regard? Issues in the efficacy and safety of psychotherapy. *J Psychopharmacol* 2008;22:3-6.

26. Deblinger E, Mannarino AP, Cohen JA, et al. Trauma-focused cognitive behavioral therapy for children: impact of the trauma narrative and treatment length. *Depress Anxiety* 2011;28:67-75.

27. Ahmad A, Larsson B, Sundelin-Wahlsten V. EMDR treatment for children with PTSD: results of a randomized controlled trial. *Nord J Psychiatry* 2007;61:349-54.

28. Ruf M, Schauer M, Neuner F, et al. Narrative exposure therapy for 7- to 16-year-olds: A randomized controlled trial with traumatized refugee children. *J Trauma Stress* 2010;23:437-45.

29. Mavridis D, Giannatsi M, Cipriani A, et al. A primer on network meta-analysis with emphasis on mental health. *Evid Based Ment Health* 2015;18:40-6.

30. Zhang Y, Zhou X, James AC, et al. Comparative efficacy and acceptability of psychotherapies for acute anxiety disorders in children and adolescents: study protocol for a network meta-analysis. *BMJ Open* 2015;5:e008572.

31. Zhou X, Hetrick SE, Cuijpers P, et al. Comparative efficacy and acceptability of psychotherapies for depression in children and adolescents: a systematic review and network meta-analysis. *World Psychiatry* 2015;14:207-22.



For peer review only - http://bmjopen.bmj.com/site/about/guidelines.xhtml

32. Carrion VG, Weems CF, Ray R, et al. Toward an empirical definition of pediatric PTSD: the phenomenology of PTSD in youth. *J Am Acad Child Adolesc Psychiatry* 2002;41:166-73.

33. Scheeringa M, Zeanah C, Myers L, et al. Predictive validity in a prospective follow-up of PTSD in preschool children. *J Am Acad Child Adolesc Psychiatry* 2005;44:899-906.

34. National Collaborating Centre for Mental Health (UK). Post-traumatic stress disorder: The management of PTSD in adults and children in primary and secondary care. Gaskell, 2005.

35. American Psychiatric Association (APA). Diagnostic and statistical manual of mental disorders (DSM-III). 3rd edn. Washington DC: American Psychiatric Association, 1980.

36. American Psychiatric Association (APA). Diagnostic and statistical manual of mental disorders (DSM-III-R). 3rd revised edn. Washington DC: American Psychiatric Association, 1987.

37. American Psychiatric Association (APA). Diagnostic and statistical manual of mental disorders (DSM-IV). 4th edn. Washington DC: American Psychiatric Association, 1994.

38. Americ an Psychiatric Association (APA). Diagnostic and statistical manual of mental disorders (DSM-IV-TR). 4th edn. Washington DC: American Psychiatric Association, 2000.

39. World Health Organization (WHO). The ninth revision of the international classification of diseases and related health problems (ICD-9). Geneva: World Health Organization, 1978.

40. World Health Organization (WHO). The tenth revision of the international classification of diseases and related health problems (ICD-10). Geneva: World Health Organization, 1992.

41. Nader KO, Newman E, Weathers FW, et al. Clinician-Administered PTSD scale for children and adolescents for DSM-IV (CAPS-CA). Los Angeles, USA: National Center for PTSD & UCLA Trauma Psychiatry Program collaboration, 1998.

42. Pynoos RS, Rodriguez N, Steinberg A, et al. The UCLA PTSD Reaction Index for DSM-IV (Revision 1). Los



For peer review only

For peer review only - http://bmjopen.bmj.com/site/about/guidelines.xhtml

Angeles: UCLA Trauma Psychiatry Program, 1998.

43. Kaufman J, Birmaher B, Brent DA, et al. Schedule for affective disorders and schizophrenia for school-aged children: present and lifetime version (K-SADS-PL): initial reliability and validity data. *J Am Acad Child Adolesc Psychiatry* 1996;36:980-88.

44. Silverman WK, Albano AM. Anxiety Disorders Interview Schedule for DSM-IV: child and parent versions. San Antonio, TX: Psychological Corporation, 1996.

45. Franklin CL, Piazza V, Chelminski I, et al. Defining subthreshold PTSD in the DSM-IV literature: a look toward DSM-5. *J Nerv Ment Dis* 2015;203:574-7.

46. Foa EB, Johnson KM, Feeny NC, et al. The child PTSD symptom scale: a preliminary examination of its psychometric properties. *J Clin Child Psychol* 2001;30:376-84.

47. Horowitz M, Wilner N, Alvarez W. Impact of event scale: a measure of subjective stress. *Psychosom Med* 1979;41:209-18.

48. Cuijpers P, Weitz E, Cristea IA, Twisk J. Pre-post effect sizes should be avoided in meta-analyses. *Epidemiol Psychiatr Sci* 2017;26:364-8.

49. da Costa BR, Nüesch E, Rutjes AW, et al. Combining follow-up and change data is valid in meta-analyses of continuous outcomes: a meta-epidemiological study. *J Clin Epidemiol* 2013;66:847-55.

50. Cuijpers P, Li J, Hofmann SG, et al. Self-reported versus clinician-rated symptoms of depression as outcome measures in psychotherapy research on depression: a meta-analysis. *Clin Psychol Rev* 2010;30:768-78.

51. Higgins JPT, Sterne JAC, Savović J, et al. A revised tool for assessing risk of bias in randomized trials In: Chandler J, McKenzie J, Boutron I, Welch V (editors). Cochrane Methods. Cochrane Database of Systematic Reviews 2016, Issue 10 (Suppl 1). dx.doi.org/10.1002/14651858. CD201601.

52. Higgins JPT, Thompson SG, Deeks JJ, et al. Measuring inconsistency in meta-analyses. *BMJ* 2003;327:557-60.



For peer review only - http://bmjopen.bmj.com/site/about/guidelines.xhtml

For peer review only

53. Furukawa TA, Barbui C, Cipriani A, et al. Imputing missing standard deviations in meta-analyses can provide accurate results. *J Clin Epidemiol* 2006;59:7-10.

54. Brooks SP, Gelman A. Alternative methods for monitoring convergence of iterative simulations. J *Comput Graph Stat* 1998;7:434-45.

55. Chaimani A, Higgins JP, Mavridis D, et al. Graphical tools for network meta-analysis in STATA. *PLoS One* 2013;8:e76654.

56. Dias S, Welton NJ, Caldwell DM, et al. Checking consistency in mixed treatment comparison meta-analysis. *Stat Med* 2010;29:932-44.

57. Salanti G, Ades AE, Ioannidis JP. Graphical methods and numerical summaries for presenting results from multiple-treatment meta-analysis: an overview and tutorial. *J Clin Epidemiol* 2011;64:163-71.

58. Salanti G, Del Giovane C, Chaimani A, et al. Evaluating the quality of evidence from a network meta-analysis. *PLoS One* 2014;9:e99682.

For peer review only



For peer review only - http://bmjopen.bmj.com/site/about/guidelines.xhtml

**Tables**

**Table 1 Description of Psychotherapeutic interventions and control condition**

| Interventions | Abbreviation | Description |
|---|---|---|
| ***Psychotherapeutic Intervention:*** | | |
| Trauma-focused cognitive-behavioral therapy | TF-CBT | CBT is a combination of cognitive and behavioral techniques. It also involves additional techniques such as relaxation training, affective modulation skills, and enhancement of future safety and development. TF-CBT is a CBT program that involves a trauma focus, which is usually performed through exposure or cognitive processing of thoughts related to the trauma. |
| Non-trauma-focused cognitive-behavioral therapy | Non-TF-CBT | Non-TF-CBT is a CBT program that focuses on teaching skills for the reduction of anxiety. These treatments utilise procedures that directly target the person's beliefs and behaviors rather than the discussions of specific traumas. |
| Cognitive therapy | CT | CT mainly uses cognitive restructuring training, which aims at examining youths' automatic thoughts and core schemas and evaluating the accuracy and affective consequences of their views. They aim to teach youths to engage in "rational" thinking about themselves, the traumatic incident, and the world. |
| Behavioral therapy | BT | BT uses some form of behavioral training, especially for exposure -based therapy and narrative therapy, to help youth reduce trauma-related symptoms. BT is based on principles of habituation. |
| Eye movement desensitization and reprocessing | EMDR | EMDR aims to help a person reprocess their memories of a traumatic event. The therapy involves bringing distressing trauma-related images, beliefs, and bodily sensations to mind. |
| Psychodynamic therapy | DYN | Psychodynamic psychotherapy focuses on integrating the traumatic experience into the life experience of the individual as a whole. Childhood issues are often felt to be important. |
| Play therapy | PT | PT used techniques to engage participants in recreational activities to help them cope with their problems and fears. |
| Stress management | SM | SM mainly includes some form of relaxation or biofeedback |
| Supportive therapy | ST | ST is an unstructured therapy without specific psychological techniques that it helped people to ventilate their experiences and emotions and offering empathy. e.g. supportive counseling, attention control, minimal contact, active listening, common factor control, nonspecific control |
| ***Control conditions:*** | | |
| Treatment-as-usual | TAU | TAU is often described as "usual care" or "usual community treatment" in trials, which may include any components of psychotherapy or pharmacotherapy for PTSD. It is not considered to be structured intervention but may have some treatment effects. |
| Waitlist | WL | WL is a control condition in which the participants receive no active treatment during the study but are informed that they can receive one after the study period is over. |
| No-treatment | NT | NT is a control condition in which the participants receive no active treatment during the study and in which they do not expect to receive such after the study is over. |



For peer review only - http://bmjopen.bmj.com/site/about/guidelines.xhtml

**Table 2 Hierarchy of PTSD symptom severity measurement scales**

| Hierarchy | PTSD symptom severity rating scales | Abbreviation |
|---|---|---|
| 1 | UCLA Posttraumatic Stress Disorder Reaction Index | UCLA PTSD Index |
| 2 | Child PTSD Symptoms Scale | CPSS |
| 3 | Clinician-Administered PTSD Scale/ Clinician Administered PTSD Scale-Child and Adolescent Version | CAPS/ CAPS-CA |
| 4 | Impact of Events Scale/ The Children's Revised Impact of Events Scale | IES/ CRIES |
| 5 | Parent report of post-traumatic symptoms/Child report of post-traumatic symptoms | PROPS/CROPS |
| 6 | Kiddie-Schedule for Affective Disorders and Schizophrenia | K-SADS |
| 7 | Trauma Symptom Checklist for Children | TSCC |
| 8 | Posttraumatic Cognitions Inventory/Child post-traumatic cognitions inventory | PTCI/CPTCI |
| 9 | Harvard Trauma Questionnaire | HTQ |
| 10 | Post traumatic Stress Scale | PSS |
| 11 | Child Post-Traumatic Stress-Reaction Index | CPTS-RI |
| 12 | The Preschool Age Psychiatric Assessment | PAPA |
| 13 | Anxiety Disorders Interview Schedule | ADIS |

For peer review only



For peer review only - http://bmjopen.bmj.com/site/about/guidelines.xhtml

**The full search strategy for database**

The following explicit search strategy with filters of clinical study will be applied: Condition = [(post-traumatic* or PTSD or PTSS or posttrauma* or trauma* or peritrauma* or peri-trauma* or psychotraumatology or “stress disorder*” or “stress reactions” or “sexually abus*” or “sexual abus*” or “sexually assault*” or “sexual assault*” or “physical abuse*” or “physically abuse*” or “child abuse” or “children abuse” or “natural disaster” or refugee or war or violence or maltreat* or mistreat or acciden* or tsunami* or hurricane* or earthquake or tornado)] AND Intervention = (“trauma focused” or trauma-specific or cogniti* or behavio* or CBT or “eye movement desensitization and reprocessing” or EMDR or EMD or “stress management” or “stress inoculation training” or mindfulness or “art therapy” or “play therapy” or hypnotherapy or biofeedback or counsel* or supportive or interpersonal or bibliotherapy or narrative or narration or exposure* or psychoeducation or “family treatment” or “family therapy” or meditation or relaxation or “problem solving” or “school-based intervention” or “school-based treatment” or “mind-body skills" or “seeking safety” or “mental health” or “writing for recovery” or “psychosocial intervention” or “child centered” or psychodynamic or psychodrama or desensitization or psychological or psychotherap*) AND Age = (adolesc* or boy* or girl* or child* or infant* or juvenil* or minors or pediatri* or paediatri* or pubescen* or puberty or student* or teen* or young or youth* or school* or high-school or preschool* or pre-school* or class*).

The following Medical Subject Headings (MeSH) will also be searched: “Stress Disorders” AND “Psychotherapy” AND (“child” OR“ adolescent”).

For peer review only

**PRISMA-P (Preferred Reporting Items for Systematic review and Meta-Analysis Protocols) 2015 checklist: recommended items to address in a systematic review protocol***

| Section and topic | Item No | Checklist item | Reported on Page # |
|---|---|---|---|
| **ADMINISTRATIVE INFORMATION** | | | |
| Title: | | | |
| Identification | 1a | Identify the report as a protocol of a systematic review | 1 |
| Update | 1b | If the protocol is for an update of a previous systematic review, identify as such | |
| Registration | 2 | If registered, provide the name of the registry (such as PROSPERO) and registration number | 4 |
| Authors: | | | |
| Contact | 3a | Provide name, institutional affiliation, e-mail address of all protocol authors; provide physical mailing address of corresponding author | 1 |
| Contributions | 3b | Describe contributions of protocol authors and identify the guarantor of the review | 17 |
| Amendments | 4 | If the protocol represents an amendment of a previously completed or published protocol, identify as such and list changes; otherwise, state plan for documenting important protocol amendments | |
| Support: | | | |
| Sources | 5a | Indicate sources of financial or other support for the review | 17 |
| Sponsor | 5b | Provide name for the review funder and/or sponsor | 17 |
| Role of sponsor or funder | 5c | Describe roles of funder(s), sponsor(s), and/or institution(s), if any, in developing the protocol | 17 |
| **INTRODUCTION** | | | |
| Rationale | 6 | Describe the rationale for the review in the context of what is already known | 6-7 |
| Objectives | 7 | Provide an explicit statement of the question(s) the review will address with reference to participants, interventions, comparators, and outcomes (PICO) | 7-8 |
| **METHODS** | | | |
| Eligibility criteria | 8 | Specify the study characteristics (such as PICO, study design, setting, time frame) and report characteristics (such as years considered, language, publication status) to be used as criteria for eligibility for the review | 8-11 |
| Information sources | 9 | Describe all intended information sources (such as electronic databases, contact with study authors, trial registers or other grey literature sources) with planned dates of coverage | 12 |
| Search strategy | 10 | Present draft of search strategy to be used for at least one electronic database, including planned limits, such that it could be | Supplementary |

For peer review only

| | | repeated | material |
|---|---|---|---|
| Study records: | | | |
| Data management | 11a | Describe the mechanism(s) that will be used to manage records and data throughout the review | 12 |
| Selection process | 11b | State the process that will be used for selecting studies (such as two independent reviewers) through each phase of the review (that is, screening, eligibility and inclusion in meta-analysis) | 12-13 |
| Data collection process | 11c | Describe planned method of extracting data from reports (such as piloting forms, done independently, in duplicate), any processes for obtaining and confirming data from investigators | 13 |
| Data items | 12 | List and define all variables for which data will be sought (such as PICO items, funding sources), any pre-planned data assumptions and simplifications | 13 |
| Outcomes and prioritization | 13 | List and define all outcomes for which data will be sought, including prioritization of main and additional outcomes, with rationale | 10-11 |
| Risk of bias in individual studies | 14 | Describe anticipated methods for assessing risk of bias of individual studies, including whether this will be done at the outcome or study level, or both; state how this information will be used in data synthesis | 13 |
| Data synthesis | 15a | Describe criteria under which study data will be quantitatively synthesised | 13-14 |
| | 15b | If data are appropriate for quantitative synthesis, describe planned summary measures, methods of handling data and methods of combining data from studies, including any planned exploration of consistency (such as $I^2$, Kendall's $\tau$) | 14 |
| | 15c | Describe any proposed additional analyses (such as sensitivity or subgroup analyses, meta-regression) | 14-15 |
| Meta-bias(es) | 16 | If quantitative synthesis is not appropriate, describe the type of summary planned | 15 |
| Confidence in cumulative evidence | 17 | Specify any planned assessment of meta-bias(es) (such as publication bias across studies, selective reporting within studies) | 15 |

*** It is strongly recommended that this checklist be read in conjunction with the PRISMA-P Explanation and Elaboration (cite when available) for important clarification on the items. Amendments to a review protocol should be tracked and dated. The copyright for PRISMA-P (including checklist) is held by the PRISMA-P Group and is distributed under a Creative Commons Attribution Licence 4.0.**

*From: Shamseer L, Moher D, Clarke M, Ghersi D, Liberati A, Petticrew M, Shekelle P, Stewart L, PRISMA-P Group. Preferred reporting items for systematic review and meta-analysis protocols (PRISMA-P) 2015: elaboration and explanation. BMJ. 2015 Jan 2;349(jan02 1):g7647.*

For peer review only

BMJ Open

# Comparative Efficacy and Acceptability of Psychotherapies for Post-traumatic Stress Disorder in Children and Adolescents: Study Protocol for a Systematic Review and Network Meta-Analysis

| | |
|---|---|
| Journal: | *BMJ Open* |
| Manuscript ID | bmjopen-2017-020198.R2 |
| Article Type: | Protocol |
| Date Submitted by the Author: | 25-Jan-2018 |
| Complete List of Authors: | Zhang, Yuqing; The First Affiliated Hospital of Chongqing Medical University, Department of Neurology<br>Zhou, Xinyu; The First Affiliated Hospital of Chongqing Medical University, Department of Psychiatry<br>Yang, Lining; The First Affiliated Hospital of Chongqing Medical University, Department of Neurology<br>Hetrick, Sarah; University of Melbourne, Centre for Youth Mental Health; University of Auckland, Department of Psychological Medicine<br>Weisz, John; Harvard University, Department of Psychology<br>Cuijpers, Pim; VU University Amsterdam, Department of Clinical Psychology<br>Barth, Juergen; University Hospital and University of Zurich, Institute for Complementary and Integrative Medicine<br>Del Giovane, Cinzia; University of Bern, Institute of Primary Health Care (BIHAM)<br>Yuan, Shuai; The First Affiliated Hospital of Chongqing Medical University, Department of Neurology<br>Cohen, David; Institut des Systèmes Intelligents et de Robotiques, UPMC Univ Paris 06, Department of Child and Adolescent Psychiatry<br>Gillies, Donna; Western Sydney Local Health District, Mental Health<br>Jiang, Xiaofeng; The First Affiliated Hospital of Chongqing Medical University, Department of Neurology<br>Teng, Teng; The First Affiliated Hospital of Chongqing Medical University, Department of Neurology<br>Xie, Peng; The First Affiliated Hospital of Chongqing Medical University, Department of Neurology; Chongqing Medical University, Institute of Neuroscience and the Collaborative Innovation Center for Brain Science |
| <b>Primary Subject Heading</b>: | Mental health |
| Secondary Subject Heading: | Evidence based practice, Pharmacology and therapeutics, Paediatrics |
| Keywords: | post-traumatic stress disorder, Child & adolescent psychiatry < PSYCHIATRY, psychotherapy, network meta-analysis, systematic review |
| | |

For peer review only

# Comparative Efficacy and Acceptability of Psychotherapies for Post-traumatic Stress Disorder in Children and Adolescents: Study Protocol for a Systematic Review and Network Meta-Analysis

Yuqing Zhang,[1] Xinyu Zhou,[2] Lining Yang,[1] Sarah E. Hetrick,[3] John R. Weisz,[4] Pim Cuijpers,[5] Jürgen Barth,[6] Cinzia Del Giovane,[7] Shuai Yuan,[1] David Cohen,[8] Donna Gillies,[9] Xiaofeng Jiang,[1] Teng Teng,[1] Peng Xie[1]

[1] Department of Neurology, The First Affiliated Hospital of Chongqing Medical University, Chongqing, China; Institute of Neuroscience and the Collaborative Innovation Center for Brain Science, Chongqing Medical University, Chongqing, China

[2] Department of Psychiatry, The First Affiliated Hospital of Chongqing Medical University, Chongqing, China

[3] Department of Psychological Medicine, University of Auckland, and the Centre of Youth Mental Health, University of Melbourne, Melbourne, Australia

[4] Department of Psychology, Harvard University, Cambridge, MA, USA

[5] Department of Clinical, Neuro and Developmental Psychology, Amsterdam Public Health research institute, Vrije Universiteit Amsterdam, The Netherlands

[6] Institute for Complementary and Integrative Medicine, University Hospital and University of Zurich, Zurich, Switzerland

[7] Institute of Primary Health Care (BIHAM), University of Bern, Switzerland

[8] Department of Child and Adolescent Psychiatry, Hôpital Pitié–Salpétrière, Institut des Systèmes Intelligents et Robotiques, Université Pierre et Marie Curie, Paris, France

[9] Western Sydney Local Health District - Mental Health, Westmead, Australia

YZ, XZ and LY contributed equally to this work.

**Direct correspondence to:**

Prof Peng Xie, Department of Neurology, The First Affiliated Hospital of Chongqing Medical University, Yuzhong District, Chongqing, China; E-mail: xiepeng973@126.com.

Keywords: post-traumatic stress disorder, child, adolescent, psychotherapy, network meta-analysis, systematic review



For peer review only - http://bmjopen.bmj.com/site/about/guidelines.xhtml

Word count: 3354.

For peer review only



For peer review only - http://bmjopen.bmj.com/site/about/guidelines.xhtml

**ABSTRACT**

**Introduction**: Post-traumatic stress disorder (PTSD) is common among children and adolescents who are exposed to trauma, and it is often associated with significant negative impacts on their psychosocial functioning and quality of life. Many types of psychotherapies have been found to be effective for PTSD in children and adolescents. However, due to the lack of direct comparisons between different psychotherapies, the hierarchy of treatment efficacy is still unclear. Therefore, we plan to conduct a systematic review and network meta-analysis to evaluate the efficacy and acceptability of various types of psychotherapies for PTSD in children and adolescents.

**Methods and analysis**: A systematic search will be conducted among eight electronic databases, including PubMed, Cochrane, EMBASE, Web of Science, PsycINFO, CINAHL, PILOTS and ProQuest Dissertations, from inception to October 2017. Randomised controlled trials (RCTs), regardless of language, publication year, and publication type, comparing any psychotherapies for PTSD to any control condition or alternative treatment in children and adolescents (18 years old or less) diagnosed with full or subclinical PTSD will be included. Study duration and the number of treatment sessions will not be limited. The primary outcome will be PTSD symptom severity at post-treatment as measured by a rating scale reported by the child, parent, or a clinician. The secondary outcomes will include (1) efficacy at follow-up; (2) acceptability (all-cause discontinuation); (3) anxiety symptom severity; (4) depressive symptom severity; and (5) quality of life and functional improvement. Bayesian network meta-analyses for all relative outcome measures will be performed. We will conduct subgroup and sensitivity network meta-analyses to determine whether the findings are affected by study characteristics. The quality of the evidence contributing to network estimates of the primary outcome will be evaluated by the GRADE framework.

**Ethics and dissemination:** No ethical issues are foreseen. The results will be published in a peer-reviewed journal, which will be disseminated electronically and in print. This network meta-analysis may be updated to inform and guide the clinical management of PTSD in children and adolescents.



For peer review only

For peer review only - http://bmjopen.bmj.com/site/about/guidelines.xhtml

**Trial registration number:** PROSPERO CRD42016051786.

For peer review only



For peer review only - http://bmjopen.bmj.com/site/about/guidelines.xhtml

**Strengths and limitations of this study**

1. Bayesian network meta-analysis can simultaneously compare various types of treatments by integrating all the best evidence (direct and indirect evidence) to estimate the interrelations across all treatments and establish a treatment hierarchy for psychotherapies for post-traumatic stress disorder in children and adolescents.

2. A number of outcomes will be used to comprehensively assess efficacy at post-treatment and follow-up, acceptability, anxiety and depressive symptom severity, quality of life and functional improvement.

3. This study will help to guide clinical decision-making regarding the relative efficacy of various types of psychotherapy, and various delivery modalities, in the treatment of post-traumatic stress disorder in children and adolescents.

4. Subgroup and sensitivity network meta-analyses will help to find potential moderators that affect the efficacy of psychotherapies.

5. The limitations of included studies will be assessed using the Cochrane Collaboration's Risk of bias' 2.0 tool and the quality of evidence for network estimates of the primary outcome will be evaluated by the GRADE framework.

For peer review only



For peer review only - http://bmjopen.bmj.com/site/about/guidelines.xhtml

## BACKGROUND

Many children and adolescents are exposed to trauma, with more than two thirds reporting at least 1 traumatic event by 16 years of age.[1] Post-traumatic stress disorder (PTSD) is one of the most common mental disorders among children and adolescents who have experienced trauma. Data from a meta-analysis pooling 72 articles with 3563 trauma-exposed children and adolescents show that the overall rate of PTSD was 15.9%.[2] As described in the Diagnostic and Statistical Manual of Mental Disorders, 5th edition (DSM-5), PTSD diagnostic criteria includes four symptom clusters: (1) re-experiencing of the traumatic event, (2) avoidance of stimuli associated with the traumatic event, (3) negative alterations in cognition and mood, (4) hyperarousal.[3] Trauma exposure is an essential factor in order to be able to diagnose PTSD, which can include physical or sexual abuse, war or terrorism, natural or man-made disasters, witnessing domestic violence, catastrophic illnesses, vehicle or other accidents.[4,5] PTSD is correlated with various adverse consequences for children and adolescents in cognitive, emotional, social, academic, and other functional domains.[6] In addition, PTSD commonly co-occurs with other psychiatric conditions, such as major depressive disorder,[7] anxiety disorder,[7] substance abuse,[8] and attention deficit hyperactivity disorder (ADHD).[9] PTSD diagnosis is accompanied by a significant reduction in quality of life, and it is estimated that successful treatment could save 2.05 quality adjusted life years (QALYs) per child or adolescent with PTSD.[10]

Several forms of interventions have been applied to address PTSD symptoms in children and adolescents, from which psychotherapy is the mainstay of treatment.[11] Although some medications (e.g., fluoxetine, paroxetine and venlafaxine) have shown a significant but small superiority over placebo (mean effect size=0.23) in PTSD treatment for adults,[12] their use in children and adolescents is still limited because (1) the evidence of the effectiveness of pharmacological treatments for children and adolescents with PTSD is scant;[13] and, (2) medications have been associated with safety concerns in children and adolescents, including evidence of increased risk of suicidality in children and adolescents treated with antidepressants.[14,15] Therefore, clinical guidelines recommend that psychotherapy should be used



For peer review only - http://bmjopen.bmj.com/site/about/guidelines.xhtml

for the initial treatment of PTSD in children and adolescents.[16]

Since the 1990s, psychotherapies have been increasingly applied in the treatment of PTSD in children and adolescents, and recent reviews have reported encouraging results on their efficacy.[17-19] Evidence from Cochrane reviews found that psychological therapies are not only effective in preventing PTSD in children and adolescents who have undergone trauma, but also effective in treating PTSD in children and adolescents.[17,18] Many types of psychotherapies are now available, such as cognitive behavioral therapy (CBT), eye movement desensitization and reprocessing (EMDR), behavioral therapy (BT), cognitive therapy, psychodynamic therapy and play therapy. Among these, trauma-focused CBT (TF-CBT), a CBT program that uses cognitive-behavioral techniques with a trauma-focused component, is the most commonly practiced psychotherapy for children and adolescents with PTSD.[20] TF-CBT has been recommended as a first-line treatment by clinical guidelines for PTSD in children and adolescents.[16,21] However, trauma-focused therapy can be difficult for some therapists to implement,[22,23] and there are some safety concerns in clinical practice (e.g. symptoms worsening and patients ending treatment prematurely).[24,25] Two recent trials that directly compared TF-CBT with non-TF-CBT alternatives reported that both were efficacious.[23,26] Thus, whether the inclusion of a trauma-focused component is essential in CBT for PTSD in children and adolescents is still uncertain. Moreover, other therapies, such as EMDR and BT, have also been reported effective in treating PTSD symptoms in children and adolescents.[27,28] However, due to methodological limitations, conventional meta-analyses cannot simultaneously compare all these treatments. Therefore, they cannot provide a clear answer regarding the best choice for initial treatment, nor can they provide a hierarchy of these psychotherapies.

Network meta-analysis is a newly developed method for evidence synthesis, which is able to integrate direct evidence (from studies directly comparing interventions) with indirect evidence (derived from separate studies addressing a common reference condition) from multiple treatment comparisons to estimate the interrelations across all treatments.[29] This approach enables a simultaneous comparison of multiple treatments, and can provide hierarchical



For peer review only - http://bmjopen.bmj.com/site/about/guidelines.xhtml

evidence to guide clinical practice. Using this method, our group has produced meta-analyses comparing the effects of different psychotherapies for the treatment of anxiety disorder[30] and depressive disorder[31] in children and adolescents. In the present study we describe the methods to undertake a network meta-analysis to complement those previous reports that will focus on the efficacy of psychotherapies for children and adolescents with PTSD. By performing a well-designed Bayesian network meta-analysis, we aim to provide a higher level of evidence that can inform clinical guidelines for PTSD treatment in children and adolescents.

For peer review only

## METHODS

### Criteria for included studies

#### *Types of studies*

All randomised controlled trials (RCTs), including cluster-randomised trials and cross-over trials, will be included. However, only the results from the first randomisation period will be considered in the cross-over trials. For the purpose of reducing heterogeneity between trials, we will exclude quasi-randomised trials (e.g., allocation based on the last number of the date of birth) and trials in which the sample size is less than 10 per study. Study duration and the number of treatment sessions will not be limited.

#### *Types of participants*

Studies that enrolled children and adolescents, aged 18 years old or less when they were initially enrolled, will be included in this review. Given that children with significant PTSD symptoms who do not meet full criteria for a PTSD diagnosis often have comparable functional impairment to those with a PTSD diagnosis,[32-34] the clinical guideline suggested that treatment decisions should be based on symptom severity and functional impairment, rather than whether or not they have an actual PTSD diagnosis.[16] Therefore, we will apply the following broad criteria to identify the participants: (1) Full PTSD, as diagnosed according to standardised diagnostic interviews based on international classifications (the Diagnostic and Statistical Manual of Mental Disorders (DSM),[3,35-38] the International Classification of



For peer review only - http://bmjopen.bmj.com/site/about/guidelines.xhtml

Diseases (ICD) [39,40]) or validated scales for PTSD based on DSM/ ICD criteria;[41-44] (2) Subclinical/Partial PTSD, defined as patients who have experienced psychological trauma and report some subsequent PTSD symptoms in at least one of the four symptom clusters according to DSM-5 (i.e., reexperiencing, avoidance, hyperarousal, negative alterations in cognition and mood);[45] (3) Clinically significant posttraumatic stress symptoms, defined as scoring above a validated cutoff on a PTSD rating scale, such as the Kiddie Schedule for Affective Disorders and Schizophrenia (K-SADS)[42], Child PTSD Symptoms Scale (CPSS)[46] and the Impact of Event Scale (IES)[47]. We will include trials in which participants have a secondary diagnosis of comorbid general psychiatric disorders, e.g., depressive disorder, anxiety disorder, attention deficit hyperactivity disorder (ADHD) and oppositional defiant disorder (ODD). However, trials in which participants have a diagnosis of acute stress disorder (ASD) or adjustment disorder (AD) will be excluded. Studies where both adults and children/adolescents are included will be eligible for inclusion, if the data for the latter can be obtained separately. Studies where it is not clear what happened to the patients who withdrew from the study will not be excluded, and all these patients will be counted as all-cause discontinuation. Trials conducted in any treatment settings, including outpatient clinics, inpatient services, community clinics and schools will be included.

***Types of interventions***

All RCTs comparing any psychotherapy against another psychotherapy or any control condition for children and adolescents with PTSD will be included. We will view each of: trauma-focused CBT (TF-CBT), non-trauma-focused CBT (Non-TF-CBT), eye movement desensitization and reprocessing (EMDR), behavioral therapy (BT), cognitive therapy (CT), psychodynamic therapy (DYN), play therapy (PT), and any other psychotherapy as independent nodes in this network meta-analysis. Trials comparing the same type of psychotherapies, but at different delivery conditions (with or without family involvement), different delivery formats (group, individual, or group plus individual) and different delivery mediums (face-to-face, internet-based) will be considered as the same node in this network meta-analysis. Nevertheless, because TF-CBT is the most commonly studied psychotherapy and recommended by clinical guidelines as



For peer review only - http://bmjopen.bmj.com/site/about/guidelines.xhtml

first-line choice for children and adolescents with PTSD,[16,21] if data available, we will separate TF-CBT with different delivery conditions, formats, and mediums as independent nodes.

Control conditions can include waitlist (WL), non-treatment (NT), and treatment as usual (TAU). We will view each of these control conditions as independent nodes in this network meta-analysis. The detailed description of each treatment and control condition are presented in Table 1. According to the principles described in this table, two reviewers will independently perform the classification of all conditions in each trial. Any disagreements will be discussed among the review team, and any unclear information will be requested from the relevant authors.

Studies where psychotherapy is used as a combination strategy (e.g., combining psychotherapy with medication) will be excluded, because such designs make it impossible for us to estimate the effect size of each specific treatment approach. We will not exclude studies that enrolled patients who had used medications in the past, provided that their medication status was not changed for at least one month prior to study entry and for the study period.

***Types of outcome measures***

*Primary outcome*

1. Efficacy at post-treatment, as measured using the end point score from PTSD symptom severity rating scales completed by the child, parent, or a clinician.[48] When end point scores are not reported, we will use change scores (if available).[49] Where more than one scale is reported in a trial, we will extract data from the PTSD symptom scales on a hierarchy, which is based on psychometric properties and appropriateness for use with children and adolescents (Table 2). In addition, where a PTSD symptom scale is reported by different raters in a trial, self-rated outcome will be preferred, then the parent or clinician rated outcome, because self-rated outcome tends to result in more conservative effect sizes.[50] Where dichotomous efficacy outcomes, instead of continuous scores, are reported in a trial, we will contact the relevant authors to request the data we need. If they didn't respond the data will not be used.

*Secondary outcomes*

1. Efficacy at follow-up, as measured by the score from PTSD scales at the longest point of follow-up (up to 12



For peer review only - http://bmjopen.bmj.com/site/about/guidelines.xhtml

months). The selection priority of PTSD scales will be the same as for efficacy at post-treatment. Data from participants who take part in subsequent treatments (e.g., continuing psychotherapy/pharmacotherapy or booster sessions) will be excluded in the follow-up analysis.

2. Acceptability, defined as all-cause discontinuation, as measured by the proportion of patients who discontinued treatment for any cause at the end of treatment. Notably, children and adolescents may discontinue treatment for many different reasons, such as finding it difficult to adhere to long-term treatment, because symptoms worsen, or due to rapid improvement of symptoms.[30]

3. Anxiety symptoms, as measured by the end point score on anxiety symptom severity rating scales. The following scales will be used: Revised Children's Manifest Anxiety Scale (RCMAS), Spence Children's Anxiety Scale (SCAS), Multidimensional Anxiety Scale for Children (MASC), State-Trait Anxiety Inventory for Children (STAIC), Screen for Anxiety and Related Disorders (SCARED). If none of above scales are reported, other valid anxiety scales will be used. When a scale is reported by different raters in a trial, self-rated outcome will be preferred.

4. Depressive symptoms, as measured by the end point score on depressive symptom severity rating scales. The following scales will used: Children's Depression Inventory (CDI), Beck depression inventory (BDI), Mood and Feeling Questionnaire (MFQ), Children's Depression Rating Scale Revised (CDRS-R), Hamilton Depression Rating Scale (HAMD). As for anxiety, other valid depression scales will be used if none of the above scales were reported.

5. Quality of life and functional improvement (QoL/functioning). If data are available, we will extract continuous outcomes from scales of quality of life and functional improvement measured at post-treatment. The scales of quality of life include Quality of Life Enjoyment and Satisfaction Questionnaire (Q-LES-Q), Pediatric Quality of Life Inventory (QoL Child Report), the Quality of Life Inventory (QoLI), and others. The scales of functional improvement include the Children's Global Assessment Scale (CGAS), the Global Assessment Functioning (GAF), and others. When scales of quality of life and functional improvement are both reported in a trial, we will extract the data of quality of life.



For peer review only - http://bmjopen.bmj.com/site/about/guidelines.xhtml

***Search strategy***

We will identify relevant trials from systematic searches in the following electronic databases: PubMed, Cochrane, EMBASE, Web of Science, PsycINFO, CINAHL, PILOTS and ProQuest Dissertations. The timeline will be from database inception to October 2017. An example of search strategy for PubMed is included in the supplementary material. In addition, international trials registers, such as World Health Organization's trials portal, ClinicalTrials.gov and Australian New Zealand Clinical Trials Registry (ANZCTR) will be searched for ongoing trials. Furthermore, we will search the presentations at European Psychiatric Association (EPA), European College of Neuropsychopharmacology (ECNP), American Psychological Association (APA), American Psychiatric Association (APA) and European Society for Child and Adolescent Psychiatry (ESCAP) congresses and hand-search relevant key psychiatric, psychological and medical journals (including *Behavior Therapy, Child Abuse and Neglect, Child Maltreatment, Journal of Counseling and Development, Counseling Outcome Research and Evaluation, Journal of Anxiety Disorders, Journal of Mental Health Counseling, Journal of Trauma Practice, Journal of Traumatic Stress, Journal of the American Academy of Child and Adolescent Psychiatry, Journal of Consulting and Clinical Psychology, Behaviour research and therapy*). The reference lists of included trials and reviews identified from initial searches will be scanned for more relevant studies. All relevant authors will be contacted to complement the incomplete data.

**Study selection and data extraction**

***Selection of trials***

Two reviewers (YZ and LY) will independently identify potentially eligible studies from the titles and abstracts of records from the search strategies. Studies will be excluded if both reviewers consider that it does not meet eligibility criteria. Then, the full texts of these potentially eligible studies will be reviewed by the same criteria. The inter-rater reliability of the two reviewers will be calculated to detect their consistency. All disagreements will be discussed and resolved by a senior review author (PX or SEH). The references of relevant reviews and included trials will be checked



For peer review only - http://bmjopen.bmj.com/site/about/guidelines.xhtml

by YZ and LY. Where multiple publications derive from a common data set, we will select the trials in which the relevant outcomes we predefined in this protocol were reported completely. We will report the reasons for exclusion for each trial in the characteristics of excluded studies list. Finally, a flow chart will be used to present the process of trial screening in this meta-analysis.

***Risk of bias assessment***

Two reviewers (YZ and XZ) will independently assess the methodological quality of the included studies. According to the Cochrane Collaboration's Risk of bias' 2.0 tool,[51] the risk of bias will be rated as "low risk", "high risk", or "some concerns" in the following domains: (1) bias arising from the randomization process, (2) bias due to deviations from intended interventions, (3) bias due to missing outcome data, (4) bias in measurement of the outcome, (5) bias in selection of the reported result, (6) overall bias. The inter-rater reliability of the two reviewers assessing the risk of bias will also be calculated. Any disagreements will be resolved by a senior review author (PX or SEH).

***Data extraction***

A standardised data extraction form will be used by two independent reviewers (YZ and XZ) to record the relevant parameters from the original paper, including study characteristics (e.g., title, first author, publication year, publication type, publication journal, location, and sponsor), patient characteristics (e.g., diagnostic criteria for PTSD, type of trauma, severity of PTSD symptoms, comorbidities, and the number, mean age and gender of participants), intervention details (e.g., type of psychotherapy, treatment format, treatment setting, treatment duration, the number of sessions, follow-up duration, and co-interventions) and outcome measures (mean scores, number of participants, and outcome raters (i.e., self rated or observer rated) for each pre-defined outcome). A table will be used to present the main characteristics of each study included in this review. The reliability of data extraction from the reviewers will also be assessed. Any disagreements will be resolved by a senior review author (PX or SEH).

**Statistical analysis**



For peer review only - http://bmjopen.bmj.com/site/about/guidelines.xhtml

We will perform Bayesian network meta-analysis for each outcome with random-effects model in WinBUGS (version 1.4.3). In addition, as a reference for relative outcomes of network meta-analyses, conventional pairwise meta-analyses will also be performed for each outcome with a random-effects model in Stata (version 13.0). Standardised mean difference (SMD, Cohen's d) will be used as a measure of effect size where outcome is measured on different scales. For dichotomous outcome (all-cause discontinuation), the effect sizes will be calculated as odds ratios (OR). Statistical heterogeneity in each pairwise comparison will be assessed with the $I^2$ statistic and p value.[52] When the mean values or SDs of continuous outcomes are missing, we will compute values by conversion from p-values, t-values, confidence intervals or standard errors.[53] Missing continuous outcome data will be analysed using completer data.

The pooled estimates of network meta-analysis will be obtained using the Markov Chains Monte Carlo method. Two Markov chains will be run simultaneously with different arbitrarily chosen initial values. Trace plots and the Brooks-Gelman-Rubin statistic will be monitored to ensure convergence.[54] When the model is adequately convergent, the foregoing samples will be discarded. Then 100,000 subsequent simulations will be run as the posterior summaries. All results will be reported as effect sizes (SMD or OR) and their 95% credible intervals (CrI). We will assume a common heterogeneity parameter for each comparison and assess the global heterogeneity using the $I^2$ statistic with 'gemtc' package in R (version 3.2.2). Furthermore, the inconsistency between direct and indirect evidence will be evaluated, which will include the assessment of global inconsistency (by comparing the fit and parsimony of consistency and inconsistency models), local inconsistency (by calculating the difference among direct and indirect evidence in closed loops in the network)[55] and the inconsistency calculated by node splitting method (by assessing the difference between direct and indirect estimates within a particular comparison).[56] Probability values will be summarised and reported as surface under the cumulative ranking curve (SUCRA) to provide a hierarchy of the treatments.[57]

***Subgroup analysis***

Where possible, we will conduct network meta-regression of data on primary outcome, to evaluate the influence of



For peer review only - http://bmjopen.bmj.com/site/about/guidelines.xhtml

the following potential moderators: (1) age group; (2) sex ratio; (3) number of sessions; (4) sample size; (5) risk of bias; (6) trauma types (acute/single trauma vs chronic trauma); (7) diagnosis criteria (youth with a diagnosis vs with subsyndromal symptoms); (8) source of outcome measure (self-rated vs observer-rated). If the data are insufficient to conduct subgroup analyses for some moderators, we will perform sensitivity analyses by omitting specific trials from the overall analysis.

***Other analyses***

Comparison-adjusted funnel plots and Egger's test will be used for each outcome to examine whether there is dominant publication bias exist in this network meta-analysis.[56] In addition, we will evaluate the quality of evidence for primary outcome by using the GRADE framework, which characterises the quality of a body of evidence on the basis of the study limitations, imprecision, inconsistency, indirectness, and publication bias for network estimates.[58]

**Discussion**

This systematic review and network meta-analysis will comprehensively evaluate the comparative efficacy and acceptability of psychological interventions for children and adolescents with PTSD. The results of this research will provide evidence to inform a hierarchy of comparative efficacy at post-treatment, efficacy with regard to PTSD symptoms at follow-up, as well as in terms of acceptability (all-cause discontinuation), improvement of anxiety symptoms, improvement of depressive symptoms, and quality of life and functional improvement. We expect that the findings could assist patients, clinicians and healthcare providers to make a better-informed choice in treatment selection.

There are some limitations in this protocol. First, due to the fact that PTSD commonly co-occurs with other psychiatric comorbidities, we will not exclude trials in which patients with comorbidity were enrolled. This will enhance the generalizability of this study; however, it will also raise the risk of bias for outcomes. Further individual patient data meta-analysis will be helpful to investigate the influence of this factor. Second, some trials may tend to report their favourite outcomes. Although we have predefined the data selection criteria for each outcome, this selection bias could



For peer review only - http://bmjopen.bmj.com/site/about/guidelines.xhtml

For peer review only

not be completely eliminated.

**Ethics and dissemination**

This network meta-analysis will use data in the public domain. Therefore, no formal ethical assessment and informed consent are required. The results will be published in a peer-reviewed journal and disseminated electronically and in print.

For peer review only



For peer review only - http://bmjopen.bmj.com/site/about/guidelines.xhtml

**Contributors**

PX, YZ and XZ conceived the study and drafted the manuscript. SEH, JRW, PC, JB, CDG, DC, DG assisted in protocol design and revision. YZ, XZ, LY, SY, XJ, and TT participated in the search strategy development, and will carry out the most work of study selection, risk of bias assessment, and data collection. PX, and SEH will help to revolve the disagreements and check the data. CDG, YZ and XZ participated in the design of data synthesis and analysis, and will conduct the statistical analyses. All the authors have approved the publication of the protocol.

**Funding** This work is supported by National Key Research and Development Programm of China (Grant No.2017YFA0505700). The funders had no role in the protocol design; the writing of the protocol; or the decision to submit the protocol for publication.

**Competing interests** SEH is an editor of the Cochrane Common Mental Disorders Group, and an author on the Cochrane systematic review on treatments for PTSD in young people. During the last two years, David Cohen reported past consultation for or the receipt of honoraria from Otsuka, Shire, Lundbeck and Roche. DG is the primary author of two reviews of psychological therapies for children and adolescents who were diagnosed with PTSD or exposed to trauma. YZ, XZ, LY, JRW, PC, JB, CDG, SY, XJ, TT, and PX declare no competing interests.

**Provenance and peer review** Not commissioned; externally peer-reviewed.

**Open Access** This is an Open Access article distributed in accordance with the Creative Commons Attribution- Non Commercial (CC BY-NC 4.0) license, which permits others to distribute, remix, adapt, build upon this work non-commercially, and license their derivative works on different terms, provided the original work is properly cited and the use is non-commercial. See: http:// creativecommons.org/- licenses/by-nc/4.0/

For peer review only

1 Copeland WE, Keeler G, Angold A, et al. Traumatic events and post-traumatic stress in childhood. *Arch General Psychiatry* 2007;64:577–84.

2. Alisic E, Zalta AK, Van Wesel F, et al. Rates of post-traumatic stress disorder in trauma-exposed children and adolescents: meta-analysis. *Br J Psychiatry* 2014;204:335-40.

3. American Psychiatric Association. Diagnostic and statistical manual of mental disorders. 5th edn. Arlington, VA: American Psychiatric Publishing, 2013.

4. Donnelly CL. Amaya-Jackson L. Post-traumatic stress disorder in children and adolescents: epidemiology, diagnosis and treatment options. *Paediatr Drugs* 2002;4:159-70.

5. Taylor TL, Chemtob CM. Efficacy of treatment for child and adolescent traumatic stress. *Arch Pediatr Adolesc Med* 2004;158:786-91.

6. Dorsey S, McLaughlin KA, Kerns SEU, et al. Evidence Base Update for Psychosocial Treatments for Children and Adolescents Exposed to Traumatic Events. *J Clin Child Adolesc Psychol* 2017;46:303-30.

7. Dixon A, Howie P, Starling J. Trauma exposure, posttraumatic stress, and psychiatric comorbidity in female juvenile offenders. *J Am Acad Child Adolesc Psychiatry* 2005;44:798-806.

8. Lipschiz D, Rasmusson AM, Anyan W, et al. Posttraumatic stress disorder and substance use in inner city girls. *J Nervous Mental Disease* 2003;191:714-21.

9. Ford JD, Racusin R, Ellis CG, et al. Child maltreatment, other trauma exposure and posttraumatic symptomatology among children with oppositional disorder and attention deficit hyperactivity disorders. *Child Maltreat* 2000;5:205-17.

10. Gospodarevskaya E. Post-traumatic stress disorder and quality of life in sexually abused Australian children. *J Child Sex Abus* 2013;22:277-96.

11. Keeshin BR, Strawn JR. Psychological and pharmacologic treatment of youth with posttraumatic stress disorder: an



For peer review only - http://bmjopen.bmj.com/site/about/guidelines.xhtml

evidence-based review. *Child Adolesc Psychiatr Clin N Am* 2014;23:399-411,

12. Hoskins M, Pearce J, Bethell A, et al. Pharmacotherapy for post-traumatic stress disorder: systematic review and meta-analysis. *Br J Psychiatry* 2015;206:93-100.
13. Bajor LA, Ticlea AN, Osser DN. The Psychopharmacology Algorithm Project at the Harvard South Shore Program: an update on posttraumatic stress disorder. *Harv Rev Psychiatry* 2011;19:240-58.
14. U.S. Food and Drug Administration (FDA). Suicidality in children and adolescents being treated with antidepressant medications. http://www.fda.gov/drugs/drugsafety/postmarketdrugsafetyinformationforpatientsandproviders/ ucm161679.htm (accessed 2 Aug 2017).
15. Cipriani A, Zhou X, Del Giovane C, et al. Comparative efficacy and tolerability of antidepressants for major depressive disorder in children and adolescents: a network meta-analysis. *Lancet* 2016;388:881-90.
16. Cohen JA, Bukstein O, Walter H, et al. Practice Parameter for the assessment and treatment of children and adolescents with posttraumatic stress disorder. *J Am Acad Child Adolesc Psychiatry* 2010;49:414-30.
17. Gillies D, Taylor F, Gray C, et al. Psychological therapies for the treatment of post-traumatic stress disorder in children and adolescents. *Cochrane Database Syst Rev* 2012;12:CD006726.
18. Gillies D, Maiocchi L, Bhandari AP, et al. Psychological therapies for children and adolescents exposed to trauma. *Cochrane Database Syst Rev* 2016;10:CD012371.
19. Gutermann J, Schreiber F, Matulis S, et al. Psychological treatments for symptoms of posttraumatic stress disorder in children, adolescents, and young adults: a meta-analysis. *Clin Child Fam Psychol Rev* 2016;19:77-93.
20. Lenz AS, Hollenbaugh KM. Meta-analysis of trauma-focused cognitive behavioral therapy for treating PTSD and co-occurring depression among children and adolescents. *Counseling Outcome Research and Evaluation* 2015;6:18-32.
21. National Collaborating Centre for Mental Health (NCCMH). Post-traumatic stress disorder: The management of

For peer review only



For peer review only - http://bmjopen.bmj.com/site/about/guidelines.xhtml

PTSD in adults and children in primary and secondary care. National Clinical Practice Guideline Number 26. London: National Institute for Clinical Excellence (NICE), 2005.

22. Cohen J, Mannarino AP, Deblinger E. Treating Trauma and Traumatic Grief in Children and Adolescents. New York: Guilford; 2006.

23. Salloum A, Overstreet S. Grief and trauma intervention for children after disaster: exploring coping skills versus trauma narration. *Behav Res Ther* 2012;50:169-79.

24. Linden M, Schermuly-Haupt ML. Definition, assessment and rate of psychotherapy side effects. *World Psychiatry* 2014;13:306-9.

25. Nutt DJ, Sharpe M. Uncritical positive regard? Issues in the efficacy and safety of psychotherapy. *J Psychopharmacol* 2008;22:3-6.

26. Deblinger E, Mannarino AP, Cohen JA, et al. Trauma-focused cognitive behavioral therapy for children: impact of the trauma narrative and treatment length. *Depress Anxiety* 2011;28:67-75.

27. Ahmad A, Larsson B, Sundelin-Wahlsten V. EMDR treatment for children with PTSD: results of a randomized controlled trial. *Nord J Psychiatry* 2007;61:349-54.

28. Ruf M, Schauer M, Neuner F, et al. Narrative exposure therapy for 7- to 16-year-olds: A randomized controlled trial with traumatized refugee children. *J Trauma Stress* 2010;23:437-45.

29. Mavridis D, Giannatsi M, Cipriani A, et al. A primer on network meta-analysis with emphasis on mental health. *Evid Based Ment Health* 2015;18:40-6.

30. Zhang Y, Zhou X, James AC, et al. Comparative efficacy and acceptability of psychotherapies for acute anxiety disorders in children and adolescents: study protocol for a network meta-analysis. *BMJ Open* 2015;5:e008572.

31. Zhou X, Hetrick SE, Cuijpers P, et al. Comparative efficacy and acceptability of psychotherapies for depression in children and adolescents: a systematic review and network meta-analysis. *World Psychiatry* 2015;14:207-22.



For peer review only

For peer review only - http://bmjopen.bmj.com/site/about/guidelines.xhtml

32. Carrion VG, Weems CF, Ray R, et al. Toward an empirical definition of pediatric PTSD: the phenomenology of PTSD in youth. *J Am Acad Child Adolesc Psychiatry* 2002;41:166-73.

33. Scheeringa M, Zeanah C, Myers L, et al. Predictive validity in a prospective follow-up of PTSD in preschool children. *J Am Acad Child Adolesc Psychiatry* 2005;44:899-906.

34. National Collaborating Centre for Mental Health (UK). Post-traumatic stress disorder: The management of PTSD in adults and children in primary and secondary care. Gaskell, 2005.

35. American Psychiatric Association (APA). Diagnostic and statistical manual of mental disorders (DSM-III). 3rd edn. Washington DC: American Psychiatric Association, 1980.

36. American Psychiatric Association (APA). Diagnostic and statistical manual of mental disorders (DSM-III-R). 3rd revised edn. Washington DC: American Psychiatric Association, 1987.

37. American Psychiatric Association (APA). Diagnostic and statistical manual of mental disorders (DSM-IV). 4th edn. Washington DC: American Psychiatric Association, 1994.

38. Americ an Psychiatric Association (APA). Diagnostic and statistical manual of mental disorders (DSM-IV-TR). 4th edn. Washington DC: American Psychiatric Association, 2000.

39. World Health Organization (WHO). The ninth revision of the international classification of diseases and related health problems (ICD-9). Geneva: World Health Organization, 1978.

40. World Health Organization (WHO). The tenth revision of the international classification of diseases and related health problems (ICD-10). Geneva: World Health Organization, 1992.

41. Nader KO, Newman E, Weathers FW, et al. Clinician-Administered PTSD scale for children and adolescents for DSM-IV (CAPS-CA). Los Angeles, USA: National Center for PTSD & UCLA Trauma Psychiatry Program collaboration, 1998.

42. Pynoos RS, Rodriguez N, Steinberg A, et al. The UCLA PTSD Reaction Index for DSM-IV (Revision 1). Los

For peer review only



For peer review only - http://bmjopen.bmj.com/site/about/guidelines.xhtml

Angeles: UCLA Trauma Psychiatry Program, 1998.

43. Kaufman J, Birmaher B, Brent DA, et al. Schedule for affective disorders and schizophrenia for school-aged children: present and lifetime version (K-SADS-PL): initial reliability and validity data. *J Am Acad Child Adolesc Psychiatry* 1996;36:980-88.

44. Silverman WK, Albano AM. Anxiety Disorders Interview Schedule for DSM-IV: child and parent versions. San Antonio, TX: Psychological Corporation, 1996.

45. Franklin CL, Piazza V, Chelminski I, et al. Defining subthreshold PTSD in the DSM-IV literature: a look toward DSM-5. *J Nerv Ment Dis* 2015;203:574-7.

46. Foa EB, Johnson KM, Feeny NC, et al. The child PTSD symptom scale: a preliminary examination of its psychometric properties. *J Clin Child Psychol* 2001;30:376-84.

47. Horowitz M, Wilner N, Alvarez W. Impact of event scale: a measure of subjective stress. *Psychosom Med* 1979;41:209-18.

48. Cuijpers P, Weitz E, Cristea IA, Twisk J. Pre-post effect sizes should be avoided in meta-analyses. *Epidemiol Psychiatr Sci* 2017;26:364-8.

49. da Costa BR, Nüesch E, Rutjes AW, et al. Combining follow-up and change data is valid in meta-analyses of continuous outcomes: a meta-epidemiological study. *J Clin Epidemiol* 2013;66:847-55.

50. Cuijpers P, Li J, Hofmann SG, et al. Self-reported versus clinician-rated symptoms of depression as outcome measures in psychotherapy research on depression: a meta-analysis. *Clin Psychol Rev* 2010;30:768-78.

51. Higgins JPT, Sterne JAC, Savović J, et al. A revised tool for assessing risk of bias in randomized trials In: Chandler J, McKenzie J, Boutron I, Welch V (editors). Cochrane Methods. Cochrane Database of Systematic Reviews 2016, Issue 10 (Suppl 1). dx.doi.org/10.1002/14651858. CD201601.

52. Higgins JPT, Thompson SG, Deeks JJ, et al. Measuring inconsistency in meta-analyses. *BMJ* 2003;327:557-60.



For peer review only - http://bmjopen.bmj.com/site/about/guidelines.xhtml

53. Furukawa TA, Barbui C, Cipriani A, et al. Imputing missing standard deviations in meta-analyses can provide accurate results. *J Clin Epidemiol* 2006;59:7-10.

54. Brooks SP, Gelman A. Alternative methods for monitoring convergence of iterative simulations. J *Comput Graph Stat* 1998;7:434-45.

55. Chaimani A, Higgins JP, Mavridis D, et al. Graphical tools for network meta-analysis in STATA. *PLoS One* 2013;8:e76654.

56. Dias S, Welton NJ, Caldwell DM, et al. Checking consistency in mixed treatment comparison meta-analysis. *Stat Med* 2010;29:932-44.

57. Salanti G, Ades AE, Ioannidis JP. Graphical methods and numerical summaries for presenting results from multiple-treatment meta-analysis: an overview and tutorial. *J Clin Epidemiol* 2011;64:163-71.

58. Salanti G, Del Giovane C, Chaimani A, et al. Evaluating the quality of evidence from a network meta-analysis. *PLoS One* 2014;9:e99682.

For peer review only



For peer review only - http://bmjopen.bmj.com/site/about/guidelines.xhtml

**Tables**

**Table 1 Description of Psychotherapeutic interventions and control condition**

| Interventions | Abbreviation | Description |
|---|---|---|
| ***Psychotherapeutic Intervention:*** | | |
| Trauma-focused cognitive-behavioral therapy | TF-CBT | CBT is a combination of cognitive and behavioral techniques. It also involves additional techniques such as relaxation training, affective modulation skills, and enhancement of future safety and development. TF-CBT is a CBT program that involves a trauma focus, which is usually performed through exposure or cognitive processing of thoughts related to the trauma. |
| Non-trauma-focused cognitive-behavioral therapy | Non-TF-CBT | Non-TF-CBT is a CBT program that focuses on teaching skills for the reduction of anxiety. These treatments utilise procedures that directly target the person's beliefs and behaviors rather than the discussions of specific traumas. |
| Cognitive therapy | CT | CT mainly uses cognitive restructuring training, which aims at examining youths' automatic thoughts and core schemas and evaluating the accuracy and affective consequences of their views. They aim to teach youths to engage in "rational" thinking about themselves, the traumatic incident, and the world. |
| Behavioral therapy | BT | BT uses some form of behavioral training, especially for exposure -based therapy and narrative therapy, to help youth reduce trauma-related symptoms. BT is based on principles of habituation. |
| Eye movement desensitization and reprocessing | EMDR | EMDR aims to help a person reprocess their memories of a traumatic event. The therapy involves bringing distressing trauma-related images, beliefs, and bodily sensations to mind. |
| Psychodynamic therapy | DYN | Psychodynamic psychotherapy focuses on integrating the traumatic experience into the life experience of the individual as a whole. Childhood issues are often felt to be important. |
| Play therapy | PT | PT used techniques to engage participants in recreational activities to help them cope with their problems and fears. |
| Stress management | SM | SM mainly includes some form of relaxation or biofeedback |
| Supportive therapy | ST | ST is an unstructured therapy without specific psychological techniques that it helped people to ventilate their experiences and emotions and offering empathy. e.g. supportive counseling, attention control, minimal contact, active listening, common factor control, nonspecific control |
| ***Control conditions:*** | | |
| Treatment-as-usual | TAU | TAU is often described as "usual care" or "usual community treatment" in trials, which may include any components of psychotherapy or pharmacotherapy for PTSD. It is not considered to be structured intervention but may have some treatment effects. |
| Waitlist | WL | WL is a control condition in which the participants receive no active treatment during the study but are informed that they can receive one after the study period is over. |
| No-treatment | NT | NT is a control condition in which the participants receive no active treatment during the study and in which they do not expect to receive such after the study is over. |



For peer review only - http://bmjopen.bmj.com/site/about/guidelines.xhtml

**Table 2 Hierarchy of PTSD symptom severity measurement scales**

| Hierarchy | PTSD symptom severity rating scales | Abbreviation |
|---|---|---|
| 1 | UCLA Posttraumatic Stress Disorder Reaction Index | UCLA PTSD Index |
| 2 | Child PTSD Symptoms Scale | CPSS |
| 3 | Clinician-Administered PTSD Scale/ Clinician Administered PTSD Scale-Child and Adolescent Version | CAPS/ CAPS-CA |
| 4 | Impact of Events Scale/ The Children's Revised Impact of Events Scale | IES/ CRIES |
| 5 | Parent report of post-traumatic symptoms/Child report of post-traumatic symptoms | PROPS/CROPS |
| 6 | Kiddie-Schedule for Affective Disorders and Schizophrenia | K-SADS |
| 7 | Trauma Symptom Checklist for Children | TSCC |
| 8 | Posttraumatic Cognitions Inventory/Child post-traumatic cognitions inventory | PTCI/CPTCI |
| 9 | Harvard Trauma Questionnaire | HTQ |
| 10 | Post traumatic Stress Scale | PSS |
| 11 | Child Post-Traumatic Stress-Reaction Index | CPTS-RI |
| 12 | The Preschool Age Psychiatric Assessment | PAPA |
| 13 | Anxiety Disorders Interview Schedule | ADIS |

For peer review only



For peer review only - http://bmjopen.bmj.com/site/about/guidelines.xhtml

**The full search strategy for PubMed**

The following explicit search strategy with filters of clinical study will be applied: Condition = [(post-traumatic* or PTSD or PTSS or posttrauma* or trauma* or peritrauma* or peri-trauma* or psychotraumatology or “stress disorder*” or “stress reactions” or “sexually abus*” or “sexual abus*” or “sexually assault*” or “sexual assault*” or “physical abuse*” or “physically abuse*” or “child abuse” or “children abuse” or “natural disaster” or refugee or war or violence or maltreat* or mistreat or acciden* or tsunami* or hurricane* or earthquake or tornado)] AND Intervention = (“trauma focused” or trauma-specific or cogniti* or behavio* or CBT or “eye movement desensitization and reprocessing” or EMDR or EMD or “stress management” or “stress inoculation training” or mindfulness or “art therapy” or “play therapy” or hypnotherapy or biofeedback or counsel* or supportive or interpersonal or bibliotherapy or narrative or narration or exposure* or psychoeducation or “family treatment” or “family therapy” or meditation or relaxation or “problem solving” or “school-based intervention” or “school-based treatment” or “mind-body skills" or “seeking safety” or “mental health” or “writing for recovery” or “psychosocial intervention” or “child centered” or psychodynamic or psychodrama or desensitization or psychological or psychotherap*) AND Age = (adolesc* or boy* or girl* or child* or infant* or juvenil* or minors or pediatri* or paediatri* or pubescen* or puberty or student* or teen* or young or youth* or school* or high-school or preschool* or pre-school* or class*).

The following Medical Subject Headings (MeSH) will also be searched: “Stress Disorders” AND “Psychotherapy” AND (“child” OR“ adolescent”).

For peer review only

**PRISMA-P (Preferred Reporting Items for Systematic review and Meta-Analysis Protocols) 2015 checklist: recommended items to address in a systematic review protocol***

| Section and topic | Item No | Checklist item | Reported on Page # |
|---|---|---|---|
| **ADMINISTRATIVE INFORMATION** | | | |
| Title: | | | |
| Identification | 1a | Identify the report as a protocol of a systematic review | 1 |
| Update | 1b | If the protocol is for an update of a previous systematic review, identify as such | |
| Registration | 2 | If registered, provide the name of the registry (such as PROSPERO) and registration number | 4 |
| Authors: | | | |
| Contact | 3a | Provide name, institutional affiliation, e-mail address of all protocol authors; provide physical mailing address of corresponding author | 1 |
| Contributions | 3b | Describe contributions of protocol authors and identify the guarantor of the review | 17 |
| Amendments | 4 | If the protocol represents an amendment of a previously completed or published protocol, identify as such and list changes; otherwise, state plan for documenting important protocol amendments | |
| Support: | | | |
| Sources | 5a | Indicate sources of financial or other support for the review | 17 |
| Sponsor | 5b | Provide name for the review funder and/or sponsor | 17 |
| Role of sponsor or funder | 5c | Describe roles of funder(s), sponsor(s), and/or institution(s), if any, in developing the protocol | 17 |
| **INTRODUCTION** | | | |
| Rationale | 6 | Describe the rationale for the review in the context of what is already known | 6-7 |
| Objectives | 7 | Provide an explicit statement of the question(s) the review will address with reference to participants, interventions, comparators, and outcomes (PICO) | 7-8 |
| **METHODS** | | | |
| Eligibility criteria | 8 | Specify the study characteristics (such as PICO, study design, setting, time frame) and report characteristics (such as years considered, language, publication status) to be used as criteria for eligibility for the review | 8-11 |
| Information sources | 9 | Describe all intended information sources (such as electronic databases, contact with study authors, trial registers or other grey literature sources) with planned dates of coverage | 12 |
| Search strategy | 10 | Present draft of search strategy to be used for at least one electronic database, including planned limits, such that it could be | Supplementary |

For peer review only

| | | repeated | material |
|---|---|---|---|
| Study records: | | | |
| Data management | 11a | Describe the mechanism(s) that will be used to manage records and data throughout the review | 12 |
| Selection process | 11b | State the process that will be used for selecting studies (such as two independent reviewers) through each phase of the review (that is, screening, eligibility and inclusion in meta-analysis) | 12-13 |
| Data collection process | 11c | Describe planned method of extracting data from reports (such as piloting forms, done independently, in duplicate), any processes for obtaining and confirming data from investigators | 13 |
| Data items | 12 | List and define all variables for which data will be sought (such as PICO items, funding sources), any pre-planned data assumptions and simplifications | 13 |
| Outcomes and prioritization | 13 | List and define all outcomes for which data will be sought, including prioritization of main and additional outcomes, with rationale | 10-11 |
| Risk of bias in individual studies | 14 | Describe anticipated methods for assessing risk of bias of individual studies, including whether this will be done at the outcome or study level, or both; state how this information will be used in data synthesis | 13 |
| Data synthesis | 15a | Describe criteria under which study data will be quantitatively synthesised | 13-14 |
| | 15b | If data are appropriate for quantitative synthesis, describe planned summary measures, methods of handling data and methods of combining data from studies, including any planned exploration of consistency (such as $I^2$, Kendall's $\tau$) | 14 |
| | 15c | Describe any proposed additional analyses (such as sensitivity or subgroup analyses, meta-regression) | 14-15 |
| Meta-bias(es) | 16 | If quantitative synthesis is not appropriate, describe the type of summary planned | 15 |
| Confidence in cumulative evidence | 17 | Specify any planned assessment of meta-bias(es) (such as publication bias across studies, selective reporting within studies) | 15 |

*** It is strongly recommended that this checklist be read in conjunction with the PRISMA-P Explanation and Elaboration (cite when available) for important clarification on the items. Amendments to a review protocol should be tracked and dated. The copyright for PRISMA-P (including checklist) is held by the PRISMA-P Group and is distributed under a Creative Commons Attribution Licence 4.0.**

*From: Shamseer L, Moher D, Clarke M, Ghersi D, Liberati A, Petticrew M, Shekelle P, Stewart L, PRISMA-P Group. Preferred reporting items for systematic review and meta-analysis protocols (PRISMA-P) 2015: elaboration and explanation. BMJ. 2015 Jan 2;349(jan02 1):g7647.*

For peer review only
